# The protective effect of hederagenin on renal fibrosis by targeting muscarinic acetylcholine receptor

**DOI:** 10.1080/21655979.2022.2054596

**Published:** 2022-03-24

**Authors:** Wei Yang, Lijuan He

**Affiliations:** a Nephrology Department, Shanxi Traditional Chinese Medicine Institute, Shanxi, China; b Acupuncture and Moxibustion Department, Xi ‘An TCM Hospital of Encephalopathy, Xi’an City, China

**Keywords:** Hederagenin, renal fibrosis, muscarinic acetylcholine receptor

## Abstract

Hederagenin (HE) plays a protective role by inhibiting cell proliferation and ameliorating fibrosis. The current therapy for Chronic kidney disease (CKD) often result in the risks of side effects. The present study aimed to explore whether it can protect against renal fibrosis and unveil the underlying mechanism. Transforming growth factor (TGF)-β was used to induce the fibroblasts NRK-49 F for the simulation of renal fibrosis. The cell viability and expression of fibrosis-related proteins in TGF-β-treated NRK-49 F cells was, respectively, measured by Cell Counting Kit-8 (CCK-8) and western blot. After predicting the target genes of HE, M3 receptor was measured in NRK-49 F cells treated with TGF-β alone or in combination with HE. Then, M3 receptor was silenced in TGF-β-treated NRK-49 F cells for the detection of its role in proliferation and fibrosis. Muscarinic acetylcholine receptor M3 (M3 receptor) agonist pilocarpine was further added to determine the role of M3 receptor involved. HE inhibited the proliferation and fibrosis of TGF-β-treated NRK-49 F cells. M3 receptor was predicted to be a target of HE. Moreover, interference of M3 receptor improved the proliferation and fibrosis of TGF-β-treated NRK-49 F cells. Further addition of pilocarpine reversed the inhibitory effect of HE on proliferation and fibrosis of TGF-β-treated NRK-49 F cells. HE protects against renal fibrosis in NRK-49 F cells by targeting Muscarinic acetylcholine receptor, which will provide theoretical basis for the clinical use of HE for kidney-related disease treatment.

## GRAPHICAL ABSTRACT

**Figure:**
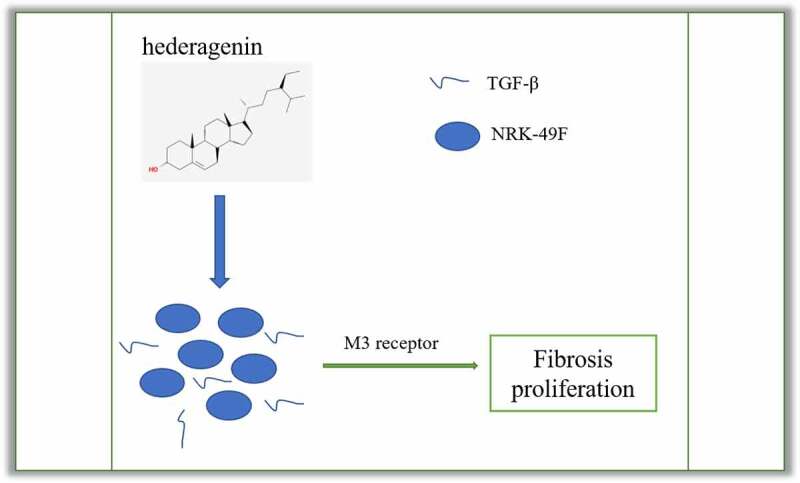

## Introduction

CKD is an irreversible change of renal structure and function characterized by glomerulosclerosis, renal tubular atrophy, and interstitial fibrosis [1]. Glomerulosclerosis is caused by endothelial injury and dysfunction, proliferation of smooth muscle cells and glomerular mesangial cells, and injury of glomerular basement membrane podocytes. Renal interstitial fibrosis is the final outcome from all chronic kidney diseases to end-stage renal disease, during the course of which normal nephrons that are able to maintain the normal kidney function are substituted by a collection of fibroblasts, myofibroblasts, and extracellular matrix.

Qi deficiency and blood stasis are considered by traditional Chinese medicine as common symptoms of renal fibrosis, while Curcumae Rhizoma has the effects of promoting Qi, breaking blood, eliminating accumulation, and relieving pain. Recently, the discovery of some drugs against CKD and the potential targets for the treatment of CKD provide novel sight for developing new treatments [^2–4^]. Natural products own widespread biological activities in treatment of some disease [^5–7^]. Studies have found that its effective component, hederagenin, can inhibit pulmonary fibrosis through RAS/JNK pathway [8], and control the proliferation of hepatic stellate cells to inhibit hepatic fibrosis [9]. Based on network pharmacology and transcriptomic analysis, the active compound hederagenin, plays a protective role in inhibiting apoptosis and fibrosis [10]. TCMSP database analysis showed that HE could interact with COA2, Nuclear receptor coactivator 2 (NCOA2) and M receptor 1, 2, and 3, among which COA2 and NCOA2 affect the development of CKD by interacting with HIF-1 [11]. It was found that overexpression of M receptor can cause cardiac and liver fibrosis [12]. M receptor and type I and III collagen (Col I, III) are involved in vascular fibrosis [12]. High expression of M receptor was also detected in patients with CKD [^13–15^]. The above evidence demonstrated the potential research value of HE in the treatment of nephropathy and inhibition of fibrosis. The purpose of this study was to explore whether HE can inhibit the development of fibrosis in CKD by regulating the expression of M receptor and regulating fibrosis signal pathway, thereby revealing the effects and mechanism of HE.

## Materials and methods

### Drug preparation and cell culture

HE (MedChemExpress; Shanghai, China) with purity >98% was dissolved in 50 mg/mL dimethyl sulfoxide (DMSO) at −80°C for experimental use. Rat renal interstitial fibroblasts NRK-49 F (Shanghai Institute for Biological Science; Shanghai, China) were maintained with 1% penicillin and 10% FBS in DMEM (high-glucose medium, HyClone, Int., USA) at 37°C under 5% CO_2_. Mycoplasma is detected at least once a week. Cells were treated with 2 ng/ml recombinant human TGF-β1 (100–21, Peprotech) for 24 h. Pilocarpine at the dose of 10 mM was used to treat the cells after TGF-β1 and HE treatment.

### Cell transfection

The siRNAs targeting M3 receptor (M3 receptor siRNA) or control (NS siRNA) were designed and synthesized by GenePharma (Shanghai, China). For transfection, the NRK-49 F cells were seeded in 96-well culture plates for M3 receptor siRNA transfection by the Lipofectamine™ 2000 Transfection reagent (Thermo Scientific, Wilmington, DE, USA). Transfected cells were cultured for 24 h prior to further experiments.

### Western blot

After NRK-49 F cells (10^5^ cells/well) received corresponding experiment treatment, total protein samples were extracted from fibroblasts using lysis buffer. The supernatant in each group was collected. After centrifugation at 12,000 rpm for 15 min at 4°C. 30 µg total protein was loaded onto 8% SDS-PAGE gels and then was electro-transferred to poly-vinylidene difluoride (PVDF) membranes (Bio-Rad, Hercules, CA, USA). Then, the membranes were sealed by 5% skimmed milk and incubated with primary antibodies for collagen I (ab260043, 1:500), collagen III (ab6310, 1:500), M1R (ab77098, 1:1000), M2R (ab109226, 1:1000), M3R (ab87199, 1:1000), TGF-β1 (ab215715, 1:1000), Smad2 (ab40855, 1:1000), Smad3 (ab40854, 1:1000), phosphorylated Smad 2 (p-Smad2, ab280888, 1:1000), p-Smad3 (AF3362, 1:1000, Affinity, USA) and glyceraldehyde 3-phosphate dehydrogenase (GAPDH) (ab8245, 1:1,000) at 4°C overnight. On the next day, secondary antibody (ab288151, 1:10,000) was used to incubate the membranes for 1 h at room temperature. The color was developed using enhanced chemiluminescence reagents, and the gray value was analyzed using ImageJ software.

### Immunofluorescent staining

NRK-49 F cells were cultured for 48 h and then washed with PBS for three times. Next, the cells were fixed with 4% paraformaldehyde for 20 min, permeabilized with 1% Triton X-100 (prepared by PBS) at room temperature for 1 h, and incubated with goat serum (Solarbio, Beijing, China) at 37°C for 0.5 h. Subsequently, the cells were incubated with collagen I antibody (1:500) at 4°C overnight, followed by incubation with fluorescence secondary antibody (1:500) and Alexa Fluor® 488-conjugated goat anti-rabbit IgG secondary antibody (Life Technologies) as a control in the dark at room temperature for 1 h. DAPI (10 mg/ml, Beyotime, Haimen, China) was used for nuclear staining. Images were captured by an Olympus microscope (Japan).

### RT-PCR assay

NRK49F cells with a density of 5 × 10^7^ L^−1^ were seeded in 6-well plates at 2 mL/well. Total RNA was extracted from transfected cells using TRIzol reagent following the manufacturer’s instructions (Invitrogen, Carlsbad, CA, USA). RNA was then reverse-transcribed into cDNA using a RevertAid First-Strand cDNA Synthesis Kit (Thermo Scientific, USA) following the manufacturer’s instructions. The PCR analysis was conducted by an ABI 7500 PCR system (ABI, USA) and the results were analyzed by software in the 7900 Real-Time PCR system. GAPDH was viewed as internal control. The primer sequences used in this study are as following: M3 receptor, Forward: 5’- ACCAACTCCTCGGCAGACAA-3’; Reverse: 5’-GCGACATCCTCTTCCGCTT −3’. GAPDH, Forward: 5’- GGTGGACCTCATGGCCTACA −3’; Reverse: 5’- CTCTCTTGCTCTCAGTATCCTTGCT −3’.

### CCK-8 assay

NRK-49 F cells (5.0 × 10^3^ cells/well) were seeded in 96-well plates for 24 h, 48 h, and 72 h. Then, Cell Counting Kit-8 (CCK-8) agent (Beyotime, China) was added to the cells. After 1 h, the absorbance was measured at 450 nm by a microplate reader (BioTek, USA) according to the manufacturer’s protocol.

### Statistical analysis

All experiments were performed at least in triplicate and analyzed using the statistical program GRAPHPAD PRISM 6 (GraphPad Prism, San Diego, CA, USA). One-way analysis of variance (ANOVA) was used for multiple comparisons among groups, followed by Turkey’s hoc test, and a P value of <0.05 meant statistical significance. The comparison between two groups were performed by t-test. Results are represented as means ± standard deviation of the mean.

## Results

Roles of HE in the proliferation, fibrosis, and downstream proteins of TGF-β in TGF-β-induced NRK-49 F cells

**Figure 1.**
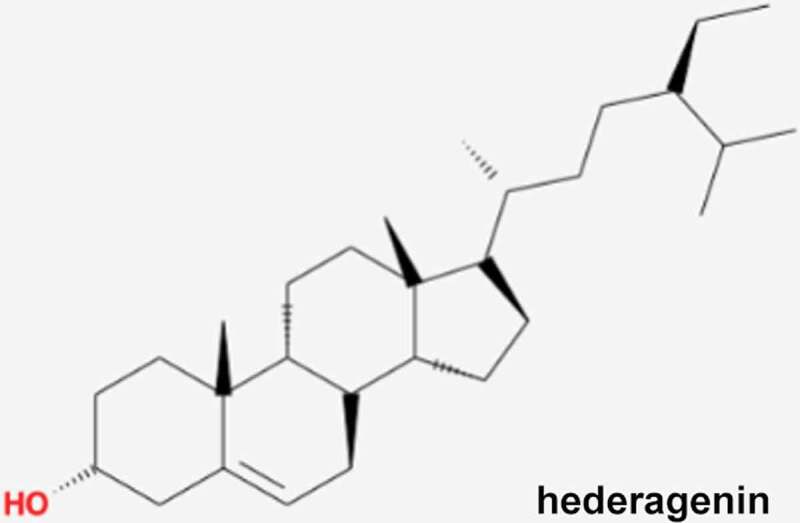
The chemical structure of HE.

**Figure 2.**
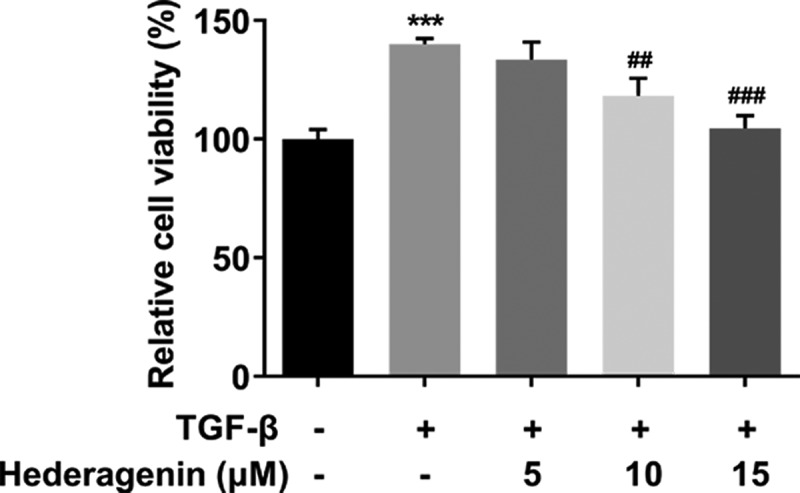
The effect of HE on the proliferation of TGF-β-induced NRK-49 F cells. The cell viability of TGF-β-induced NRK-49 F cells exposed to HE. ***P < 0.001 versus TGF-β. ^##^P < 0.01, ^###^P < 0.001 versus TGF-β + Hederagenin. Each experiment was repeated at least three times.

**Figure 3.**
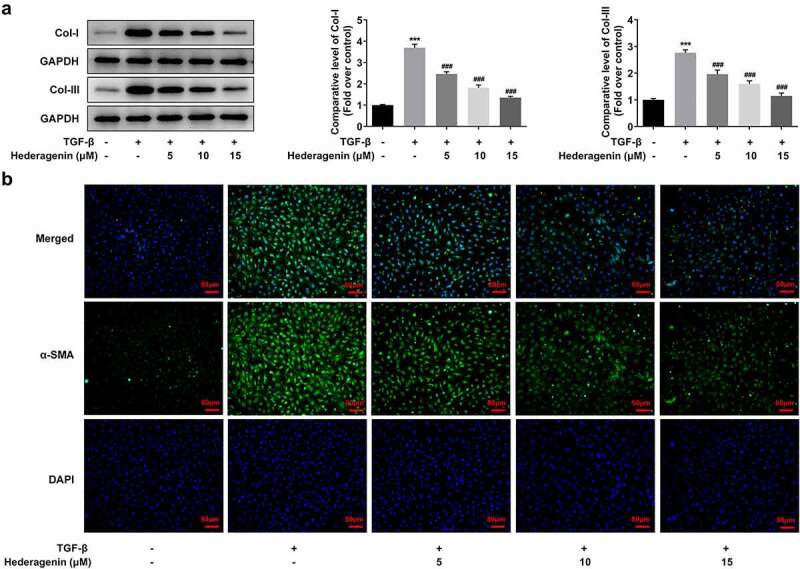
The effect of HE on the fibrosis of TGF-β-induced NRK-49 F cells. The expression of Col-I and Col-III (a) and (b) a-SMA in TGF-β-induced NRK-49 F cells exposed to HE. ***P < 0.001 versus TGF-β. ^###^P < 0.001 versus TGF-β + Hederagenin. Representative images were shown. Each experiment was repeated at least three times.

**Figure 4.**
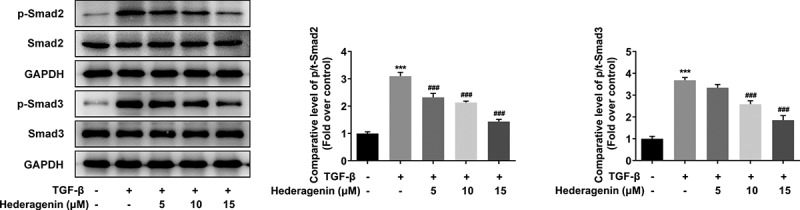
The effect of HE on the downstream proteins of TGF-β in TGF-β-induced NRK-49 F cells. ***P < 0.001 versus TGF-β. ^###^P < 0.001 versus TGF-β + Hederagenin. Representative images of western blot assay. Each experiment was repeated at least three times.

To examine the role of HE in renal fibrosis, 2 ng/ml TGF-β was used to induce the NRK-49 F cells for 24 h. The chemical structure of HE is shown in Figure 1. After the detection from CCK-8 assay kit that was used to measure NRK-49 F cell viability, we found that TGF-β-induced abnormally high cell viability of NRK-49 F cells was concentration-dependently restored by HE (Figure 2). As presented in Figure 3(a,b), the expression of Col-I, Col-III, α-SMA stimulated by TGF-β was downregulated by HE, indicating the capability of HE to alleviate renal fibrosis. In addition, the levels of downstream proteins of TGF-β, which are p-Smad2 and p-Smad3, are increased by TGF-β induction, while increased upon HE treatment (Figure 4).

Effect of HE on expression of acetylcholine receptors in TGF-β-induced NRK-49 F cells

**Figure 5.**
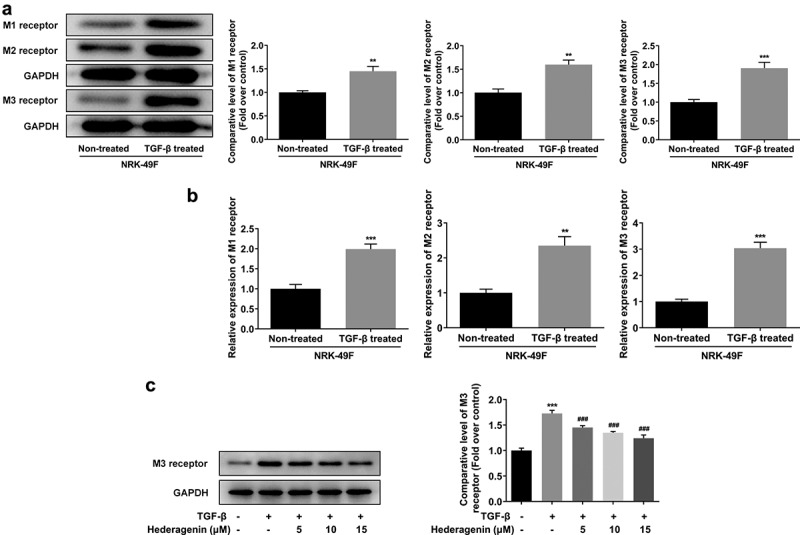
The expression of M1, M2 and M3 receptor in TGF-β-induced NRK-49 F cells. The expression of M1, M2 and M3 receptor in (a, b) NRK-49 F cells induced only by TGF-β, **P < 0.01, ***P < 0.001 versus Non-treated. and (c) in combination with HE, ***P < 0.001 versus TGF-β. ^###^P < 0.001 versus TGF-β + Hederagenin. Representative images of western blot assay. Each experiment was repeated at least three times.

TCMSP database (https://tcmspw.com/molecule.php?qn=296) predicted the related genes of HE was acetylcholine receptors M1, M2, and M3. Western blot and PCR analyses demonstrated higher levels of these receptors in TGF-β-induced NRK-49 F cells compared with those without treatment of TGF-β (Figure 5(a,b)). Interestingly, upon HE exposure, the levels of these receptors were decreased (Figure 5(c)).

M3 receptor silencing improved the proliferation, fibrosis, and downstream proteins of TGF-β in TGF-β-induced NRK-49 F cells

**Figure 6.**
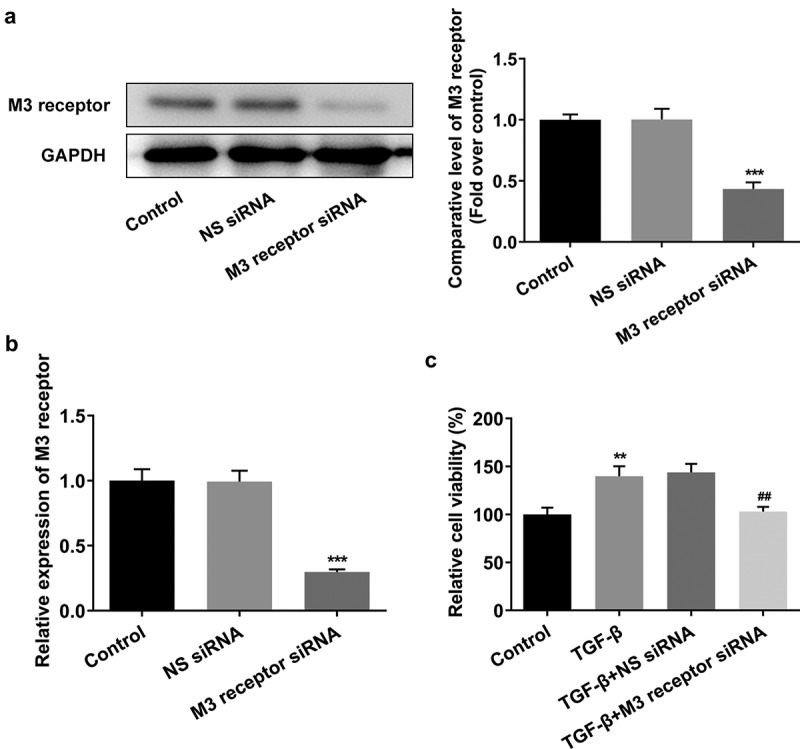
The effect of M3 receptor interference on the proliferation of TGF-β-induced NRK-49 F cells. The transfection efficiency of M3 receptor was confirmed by WB and PCR (a-b). Representative images of western blot assay. ***P < 0.001 versus NS siRNA. The cell viability of TGF-β-induced NRK-49 F cells interfered by M3 receptor (c). **P < 0.01 versus Control. ^###^P < 0.001 versus TGF-β+ NS siRNA. NS siRNA is the control group of M3 receptor siRNA. Each experiment was repeated at least three times.

**Figure 7.**
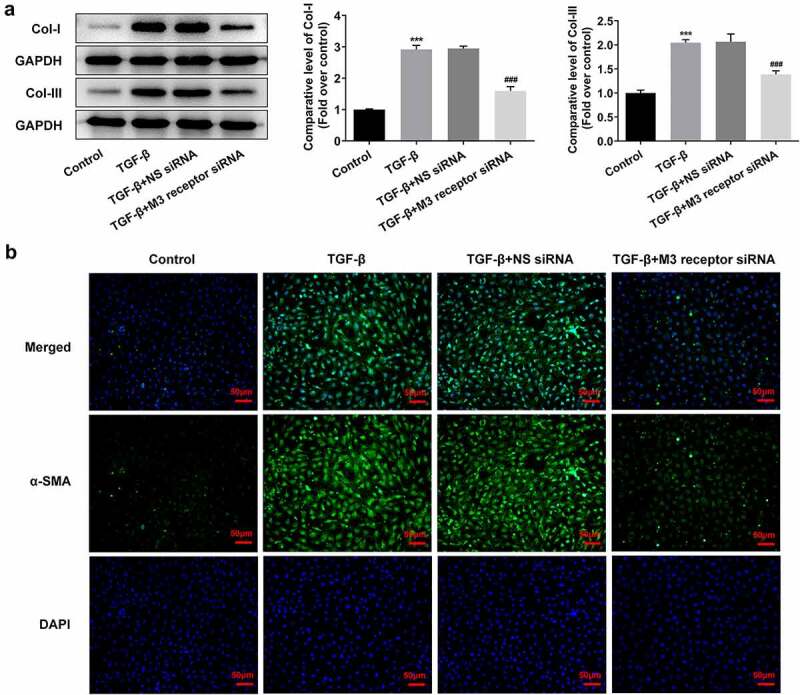
The effect of M3 receptor interference on the fibrosis of TGF-β-induced NRK-49 F cells. The expression of (a) Col-I and Col-III and (b) a-SMA in TGF-β-induced NRK-49 F cells interfered by M3 receptor. ***P < 0.001 versus Control. ^###^P < 0.001 versus TGF-β + NS siRNA. NS siRNA is the control group of M3 receptor siRNA. Representative images were shown. Each experiment was repeated at least three times.

**Figure 8.**
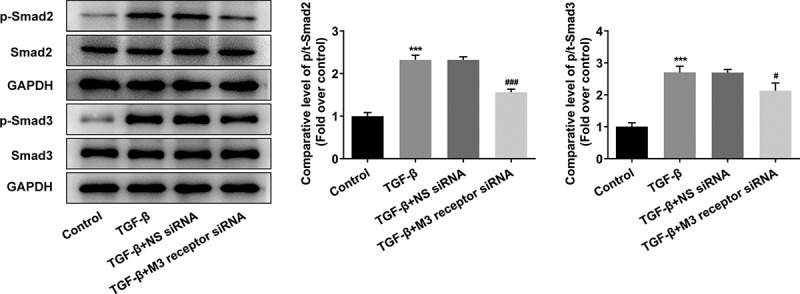
The role of M3 receptor on the downstream proteins of TGF-β in TGF-β-induced NRK-49 F cells. ***P < 0.001 versus Control. ^#^P < 0.05, ^###^P < 0.001 versus TGF-β + NS siRNA. NS siRNA is the control group of M3 receptor siRNA. Representative images of western blot assay. The experiment was repeated at least three times.

To reveal the role of M3 receptor, we silenced M3 receptor in order to observe the effects of M3 receptor on the proliferation and fibrosis of TGF-β-induced NRK-49 F cells, and the transfection efficiency of siRNA targeting M3 receptor was confirmed by Western blot and PCR (Figure 6(a)). Meanwhile, M3 receptor siRNA decreased the TGF-β-induced high viability of NRK-49 F cells (Figure 6(b)). The expression of Col-I, Col-III, and α-SMA increased by TGF-β was decreased by M3 receptor siRNA (Figure 7(a,b)). Furthermore, the presence of M3 receptor siRNA in TGF-β-induced NRK-49 F cells suppressed the expression of p-Smad2 and p-Smad3 (Figure 8). Thus, these data indicated that M3 receptor silencing improved the proliferation and fibrosis, and decreasing the activation of downstream proteins of TGF-β in TGF-β-induced NRK-49 F cells.

HE improved the renal fibrosis of TGF-β-induced NRK-49 F cells by activating M3 receptor

**Figure 9.**
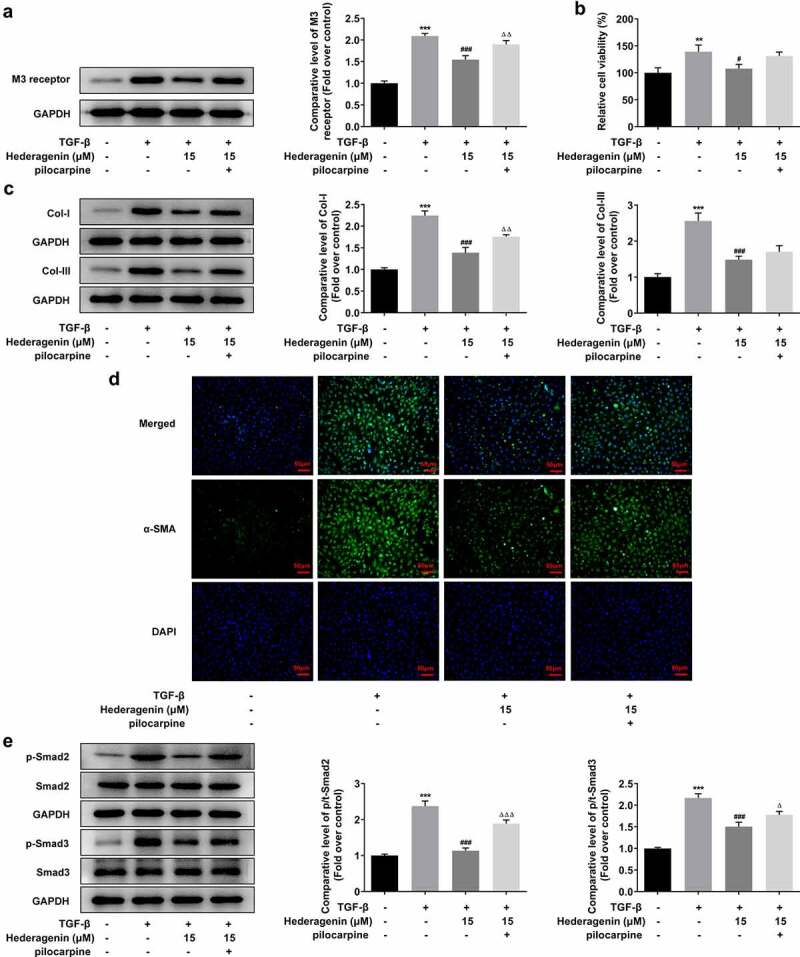
HE improves TGF-β-induced renal fibrosis via M3 receptor. (a) The expression of M3 receptor, (b) cell viability, (c) expression of Col-I and Col-III (d) a-SMA, and (e) downstream proteins of TGF-β in TGF-β-induced NRK-49 F cells exposed to HE and pilocarpine. **P < 0.01, ***P < 0.001 versus control, ^#^P < 0.05, ^###^ P < 0.001 versus TGF-β, ^ΔΔ^P<0.01 versus TGF-β + Hederagenin. Representative images were shown. Each experiment was repeated at least three times.

To determine whether HE exerted inhibitory effects on the fibrosis of TGF-β-induced NRK-49 F cells via M3 receptor, we then added M3 receptor agonist pilocarpine at the dose of 10 um on the basis of TGF-β plus HE treatment in NRK-49 F cells. Evidently, the level of M3 receptor was elevated upon pilocarpine addition (Figure 9(a)). It was obviously observed from western blot results and immunofluorescent images that the suppressive impact of HE on the cell viability and fibrosis was partially abated by pilocarpine, as evidenced by increased cell viability, Col-I and Col-III, and α-SMA expression after pilocarpine treatment (Figure 9(b-d)). Finally, pilocarpine also upregulated the levels of p-Smad2 and p-Smad3, as compared to TGF-β + Hederagenin 15uM group.

## Discussion

The intricate process of renal fibrosis is implicated in many stimulative factors and cells, especially mesenchymal cells [16]. Under the condition of inflammatory status, hypoxia or mechanical forces, fibroblasts found within the renal interstitium are activated and act as contractile myofibroblastic phenotype to impair renal function, by which renal disease can progress to more aggravated extent [17]. As we all know, renal fibrosis in CKD is elicited by persistent inflammation, and TGF-β regulates the renal fibrosis via a variety of pathways [18]. Thus, in this paper, TGF-β was chosen for the induction of renal fibrosis in fibroblasts.

HE is of great value in the amelioration of renal fibrosis. The clinical drug for renal fibrosis is easy to lead to side effects, such as renal toxicity. A study found that prednisone therapy would result in steroid-sensitive forms appearing frequently relapse and many patients have to require second-line steroid-sparing immunosuppression [19]. Natural products are considered as useful and safe, and own renal protective effects, some of which are widely used against renal disease [20]. Consistent with previous study indicating the important role of HE as an anti-proliferative agent, the TGF-β-elevated proliferation of fibroblasts was attenuated as a consequence of HE administration. The aggregation of extracellular matrix (ECM) proteins like type I and III collagen (COL I and III) can substitute the functional tissue, thereby decaying the filtration surface area of the glomeruli and triggering the occurrence of fibrosis [21,22]. It is well recognized that abnormal activation of fibroblasts and the expression of α-SMA are hallmarks of renal fibrosis [23]. In the present study, the expression of COL I and III and α-SMA was dramatically increased by TGF-β stimulation, while restored upon HE treatment, suggesting that HE imposed inhibitory effects on ECM deposition for the suppression of renal fibrosis. It has been reported that TGF-β1/Smad pathway activation can enhance the growth of cardiac fibroblasts and secretion of collagen [24]. Concurrently, the following results in our study validated this finding, as highlighted by the increased expression of p-Smad2 and p-Smad3 upon TGF-β induction in NRK-49 F cells.

The M3 muscarinic acetylcholine receptor (M3 receptor) is a prototypic member of the five GPCR subtypes (M1–M5), through which acetylcholine plays numerous physiological functions [25]. M3 receptor is involved in the proliferation of fibroblasts and collagen production and it represents a potential drug target for many human diseases [26]. Selective activation of M3 receptor reduces hepatic collagen deposition, bile ductule proliferation, and hepatic fibrosis [27]. Study showing M3 receptors on structural cells mediated neutrophilic airway inflammation in mice inhaled in cigarette smoke proposed the significant proinflammatory role of M3 receptor, and postulation that inhibition of M3 receptor alleviated the CKD by modulating the inflammation was put forward [28]. Moreover, the long acting muscarinic receptor inhibitor tiotropium bromide, is a potent agent for patients with bronchial asthma together with chronic obstructive pulmonary disease [29]. Further study suggested that this antimuscarinic drug exerted beneficial effects on remodeling processes in chronic airway disease by anti-proliferative impacts on fibroblasts and myofibroblasts [30]. Thus, in the present study, we inhibited the expression of M3 receptor by siRNA to observe whether M3 receptor participated in the suppressive effect of HE on CKD, identifying that M3 receptor silencing improved the proliferation, fibrosis, and downstream proteins of TGF-β in TGF-β-induced NRK-49 F cells. In the present study, lack of animal experiments is a limitation of this study, which will be taken into consideration in future studies. In addition, how HE modulates M3 receptor in response to TGF-β still requires deeper exploration.

## Conclusion

HE protects against renal fibrosis in NRK-49 F cells by targeting Muscarinic acetylcholine receptor, which will provide theoretical basis for the clinical use of HE for kidney-related disease treatment.

## Supplementary Material

Supplemental Material

## Data Availability

The datasets used and/or analyzed during the current study are available from the corresponding author and the first author on reasonable request.

## References

[1] Akchurin OM. Chronic kidney disease and dietary measures to improve outcomes. Pediatr Clin North Am. 2019;66(1):247–267. 30454747 10.1016/j.pcl.2018.09.007 PMC6623973

[2] Jia Y, Xu H, Yu Q, et al. Identification and verification of vascular cell adhesion protein 1 as an immune-related hub gene associated with the tubulointerstitial injury in diabetic kidney disease. Bioengineered. 2021;12(1):6655–6673. 34506229 10.1080/21655979.2021.1976540 PMC8806788

[3] Tu H, Ma D, Luo Y, et al. Quercetin alleviates chronic renal failure by targeting the PI3k/Akt pathway. Bioengineered. 2021;12(1):6538–6558. 34528858 10.1080/21655979.2021.1973877 PMC8806539

[4] Xie Z, Wei L, Chen J, et al. Calcium dobesilate alleviates renal dysfunction and inflammation by targeting nuclear factor kappa B (NF-κB) signaling in sepsis-associated acute kidney injury. Bioengineered. 2022;13(2):2816–2826. 35038964 10.1080/21655979.2021.2024394 PMC8974157

[5] Muhammad N, Steele R, Isbell TS, et al. Bitter melon extract inhibits breast cancer growth in preclinical model by inducing autophagic cell death. Oncotarget. 2017;8(39):66226–66236. 29029506 10.18632/oncotarget.19887 PMC5630406

[6] Bhattacharya S, Muhammad N, Steele R, et al. Bitter melon enhances natural killer-mediated toxicity against head and neck cancer cells. Cancer Prev Res (Phila). 2017;10(6):337–344. 28465362 10.1158/1940-6207.CAPR-17-0046 PMC5499682

[7] Bhattacharya S, Muhammad N, Steele R, et al. Immunomodulatory role of bitter melon extract in inhibition of head and neck squamous cell carcinoma growth. Oncotarget. 2016;7(22):33202–33209. 27120805 10.18632/oncotarget.8898 PMC5078086

[8] Ma W, Huang Q, Xiong G, et al. The protective effect of Hederagenin on pulmonary fibrosis by regulating the Ras/JNK/NFAT4 axis in rats. Biosci Biotechnol Biochem. 2020;84(6):1131–1138. 32024440 10.1080/09168451.2020.1721263

[9] Xu J, Wang X, Zhang H, et al. Synthesis of triterpenoid derivatives and their anti-tumor and anti-hepatic fibrosis activities. Nat Prod Res. 2020;34(6):766–772. 30445851 10.1080/14786419.2018.1499642

[10] Wu R, Dong S, Cai FF, et al. Active compounds derived from fuzheng huayu formula protect hepatic parenchymal cells from apoptosis based on network pharmacology and transcriptomic analysis. Molecules. 2019;24(2): 338. 10.3390/molecules24020338 PMC6358846 30669350

[11] Zhou T, Lin W, Lin S, et al. Association of nuclear receptor coactivators with hypoxia-inducible factor-1α in the serum of patients with chronic kidney disease. Biomed Res Int. 2020;2020:1587915. 32884936 10.1155/2020/1587915 PMC7455818

[12] Ma G, Wu X, Zeng L, et al. Association of autoantibodies against M2-muscarinic acetylcholine receptor with atrial fibrosis in atrial fibrillation patients. Cardiol Res Pract. 2019;2019:8271871. 30863630 10.1155/2019/8271871 PMC6378765

[13] Luo L, Xi C, Xu T, et al. Muscarinic receptor mediated signaling pathways in hepatocytes from CCL4 - induced liver fibrotic rat. Eur J Pharmacol. 2017;807:109–116. 28342980 10.1016/j.ejphar.2017.03.047

[14] Chen SF, Lee CL, Kuo HC. Changes in sensory proteins in the bladder urothelium of patients with chronic kidney disease and end-stage renal disease. Low Urin Tract Symptoms. 2019;11(2):O202–O208. 30198096 10.1111/luts.12240

[15] Robey RB, Ruiz OS, Baniqued J, et al. SFKs, Ras, and the classic MAPK pathway couple muscarinic receptor activation to increased Na-HCO3 cotransport activity in renal epithelial cells. Am J Physiol Renal Physiol. 2001;280(5):F844–850. 11292627 10.1152/ajprenal.2001.280.5.F844

[16] Qi R, Yang C. Renal tubular epithelial cells: the neglected mediator of tubulointerstitial fibrosis after injury. Cell Death Dis. 2018;9(11):1126. 30425237 10.1038/s41419-018-1157-x PMC6233178

[17] Nogueira A, Pires MJ, Oliveira PA. Pathophysiological mechanisms of renal fibrosis: a review of animal models and therapeutic strategies. In Vivo. 2017;31(1):1–22. 28064215 10.21873/invivo.11019 PMC5354133

[18] Meng XM, Nikolic-Paterson DJ, Lan HY. TGF-beta: the master regulator of fibrosis. Nat Rev Nephrol. 2016;12(6):325–338. 27108839 10.1038/nrneph.2016.48

[19] Vivarelli M, Massella L, Ruggiero B, et al. Minimal change disease. Clin J Am Soc Nephrol. 2017;12(2):332–345. 27940460 10.2215/CJN.05000516 PMC5293332

[20] Gao C, Liu C, Chen Y, et al. Protective effects of natural products against drug-induced nephrotoxicity: a review in recent years. Food Chem Toxicol. 2021;153:112255. 33989732 10.1016/j.fct.2021.112255

[21] Rasmussen DGK, Nielsen PM, Kasab-Oglo OY, et al. A non-invasive biomarker of type III collagen degradation reflects ischaemia reperfusion injury in rats. Nephrol Dial Transplant. 2019;34(8):1301–1309. 30535162 10.1093/ndt/gfy345

[22] Zhang C, Zhu Z, Wang G, et al. Effects of mycophenolate mofetil on renal interstitial fibrosis after unilateral ureteral obstruction in rats. J Huazhong Univ Sci Technolog Med Sci. 2003;23(3):269–270, 282. 14526430 10.1007/BF02829510

[23] Sun YB, Qu X, Caruana G, et al. The origin of renal fibroblasts/myofibroblasts and the signals that trigger fibrosis. Differentiation. 2016;92(3):102–107. 27262400 10.1016/j.diff.2016.05.008

[24] Zhang M, Pan X, Zou Q, et al. Notch3 ameliorates cardiac fibrosis after myocardial infarction by inhibiting the TGF-beta1/Smad3 pathway. Cardiovasc Toxicol. 2016;16(4):316–324. 26487518 10.1007/s12012-015-9341-z

[25] Kruse AC, Li J, Hu J, et al. Novel insights into M3 muscarinic acetylcholine receptor physiology and structure. J Mol Neurosci. 2014;53(3):316–323. 24068573 10.1007/s12031-013-0127-0 PMC3966988

[26] Zhao L, Chen T, Hang P, et al. Choline attenuates cardiac fibrosis by inhibiting p38MAPK signaling possibly by acting on M3 muscarinic acetylcholine receptor. Front Pharmacol. 2019;10:1386. 31849653 10.3389/fphar.2019.01386 PMC6900736

[27] Khurana S, Jadeja R, Twaddell W, et al. Effects of modulating M3 muscarinic receptor activity on azoxymethane-induced liver injury in mice. Biochem Pharmacol. 2013;86(2):329–338. 23707755 10.1016/j.bcp.2013.05.010 PMC3699334

[28] Kistemaker LE, van Os RP, Dethmers-Ausema A, et al. Muscarinic M3 receptors on structural cells regulate cigarette smoke-induced neutrophilic airway inflammation in mice. Am J Physiol Lung Cell Mol Physiol. 2015;308(1):L96–103. 25381025 10.1152/ajplung.00259.2014 PMC4315453

[29] Kang JY, Rhee CK, Kim JS, et al. Effect of tiotropium bromide on airway remodeling in a chronic asthma model. Ann Allergy Asthma Immunol. 2012;109(1):29–35. 22727154 10.1016/j.anai.2012.05.005

[30] Pieper MP, Chaudhary NI, Park JE. Acetylcholine-induced proliferation of fibroblasts and myofibroblasts in vitro is inhibited by tiotropium bromide. Life Sci. 2007;80(24–25):2270–2273. 17412366 10.1016/j.lfs.2007.02.034

