# Supplementary material for: The protective effect of hederagenin on renal fibrosis by targeting muscarinic acetylcholine receptor
Source: Bioengineered. 2022 Mar 24;13(4):8689–98. doi: 10.1080/21655979.2022.2054596 (PMC9161953; doi:10.1080/21655979.2022.2054596)
Supplement: Supplemental Material [file KBIE_A_2054596_SM6600.docx]

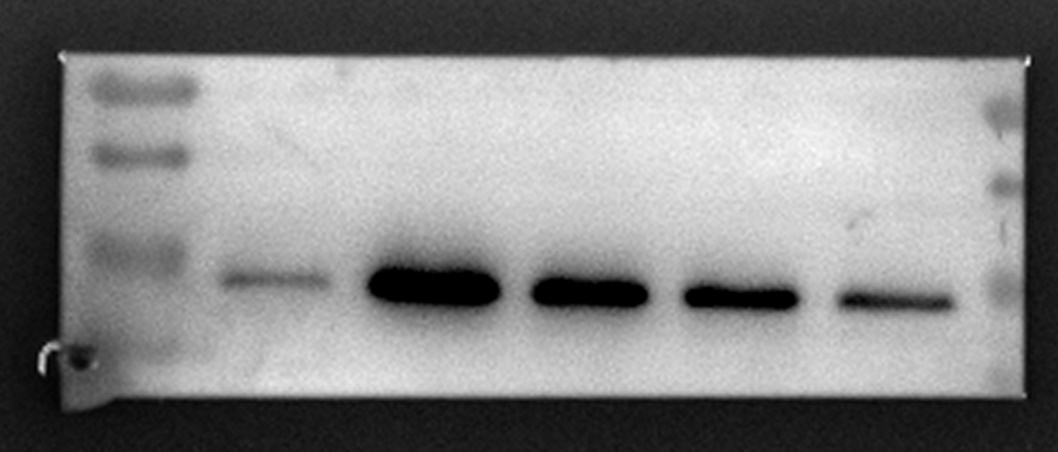


Col-Ⅰ


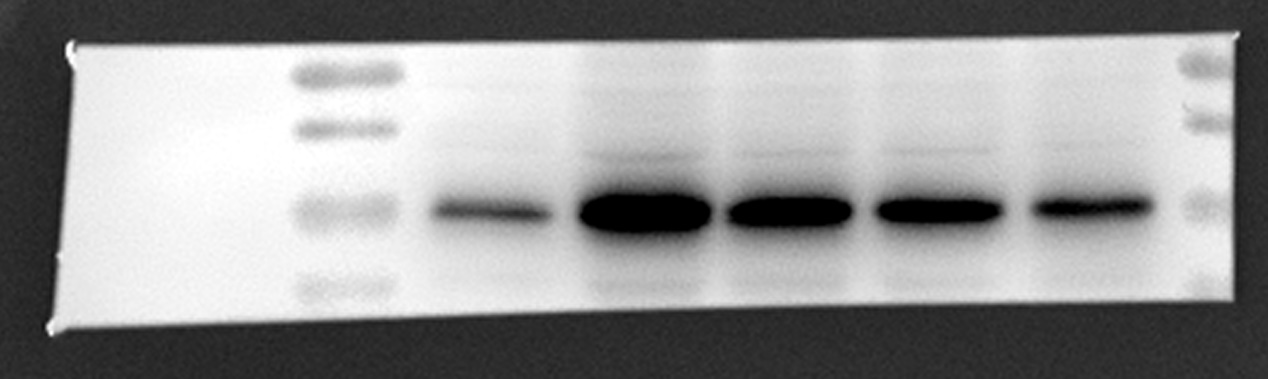


Col-Ⅲ


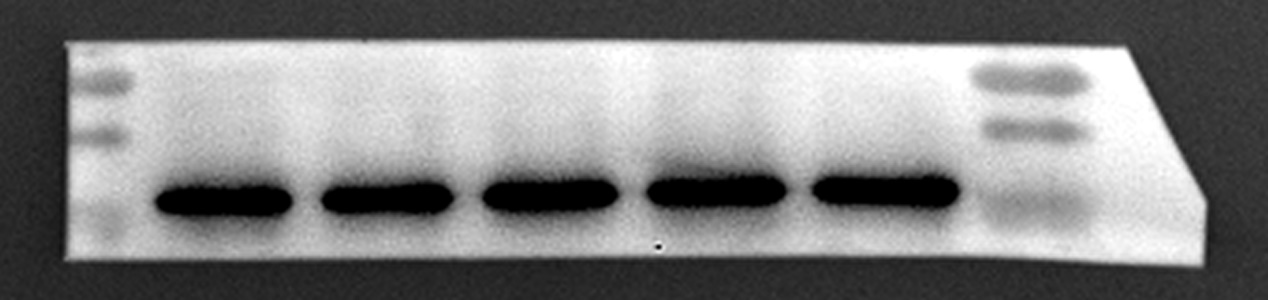


gapdh-1


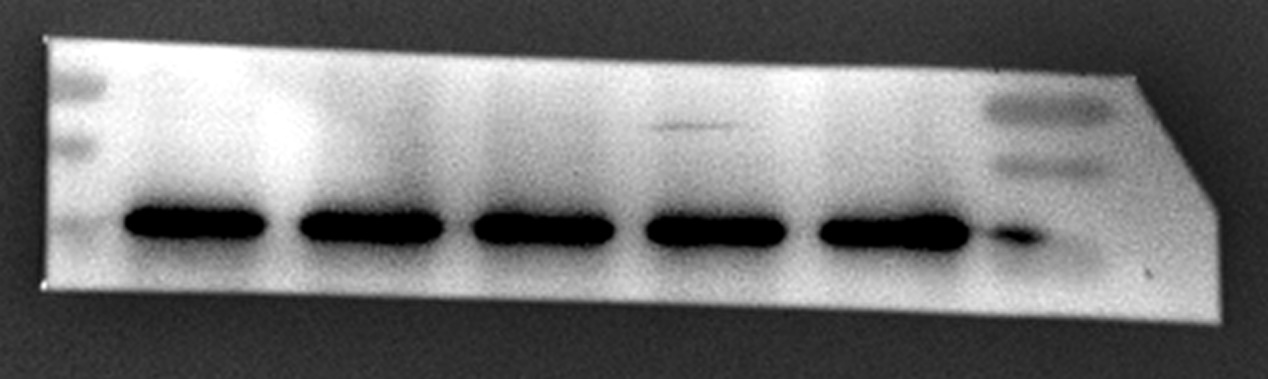


Gapdh


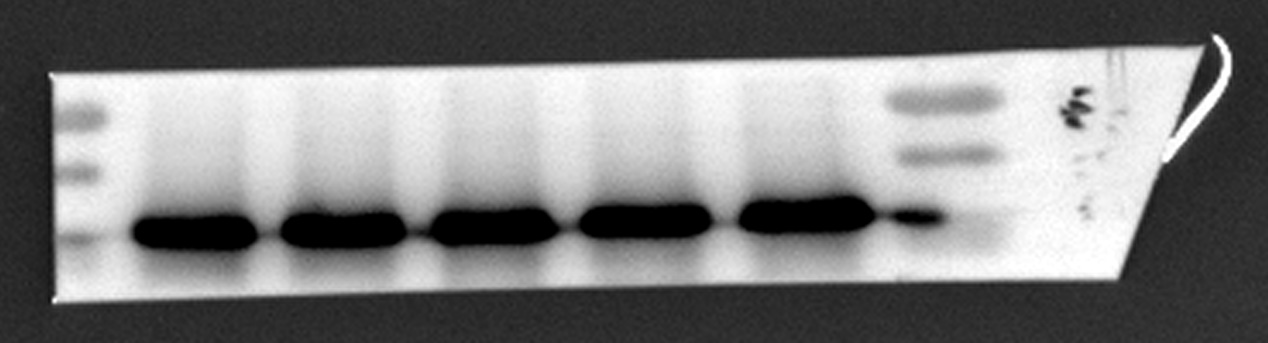


GAPDH


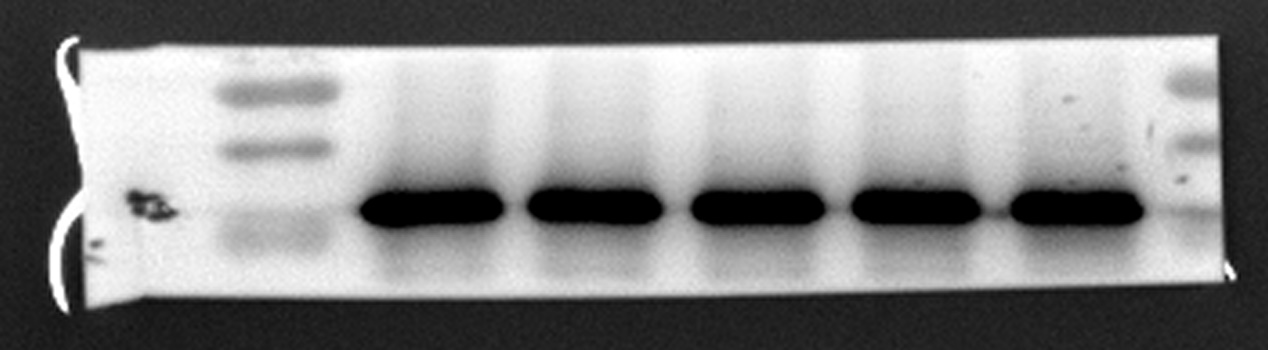


GAPDH


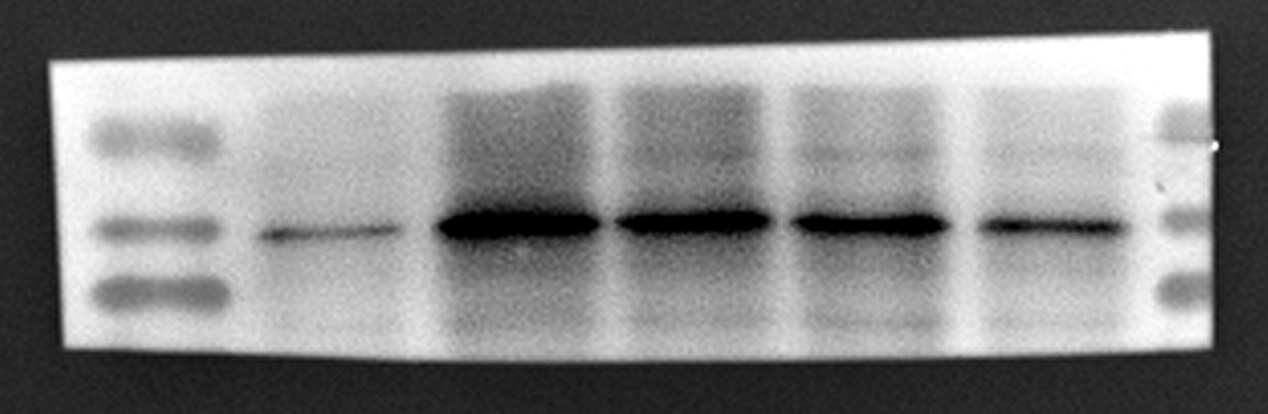


p-Smad2


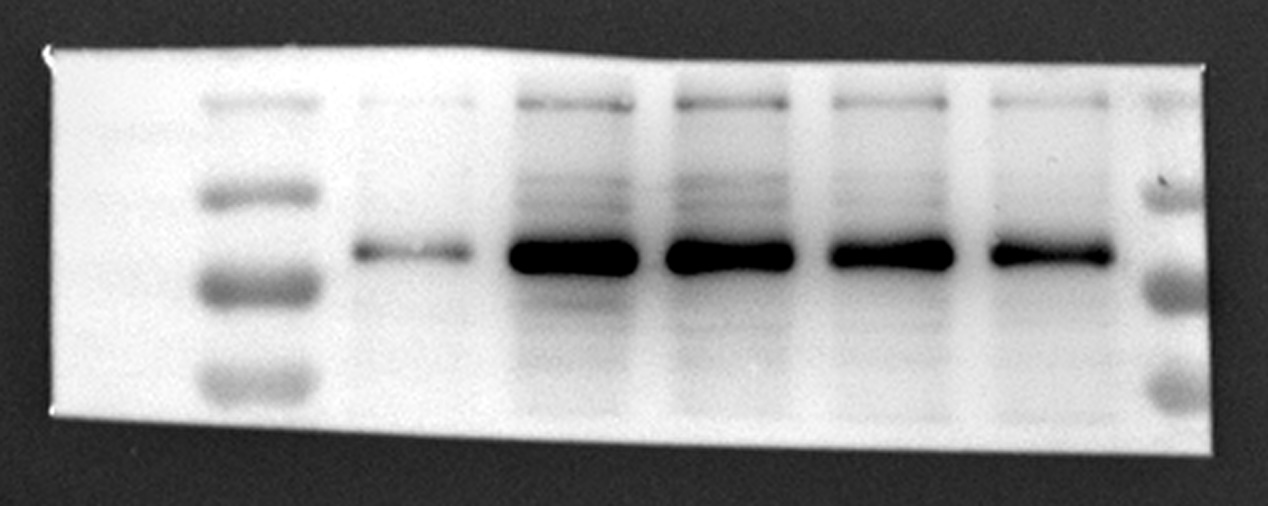


p-Smad3


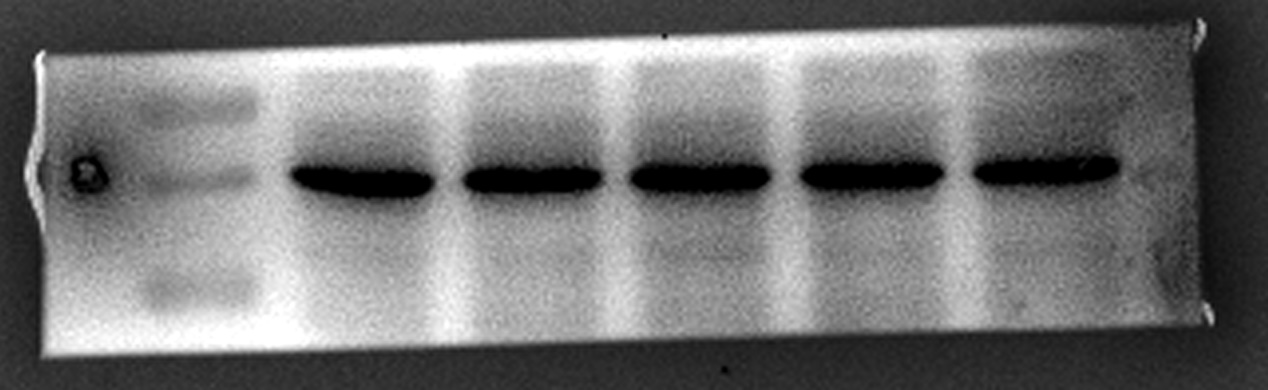


Smad2


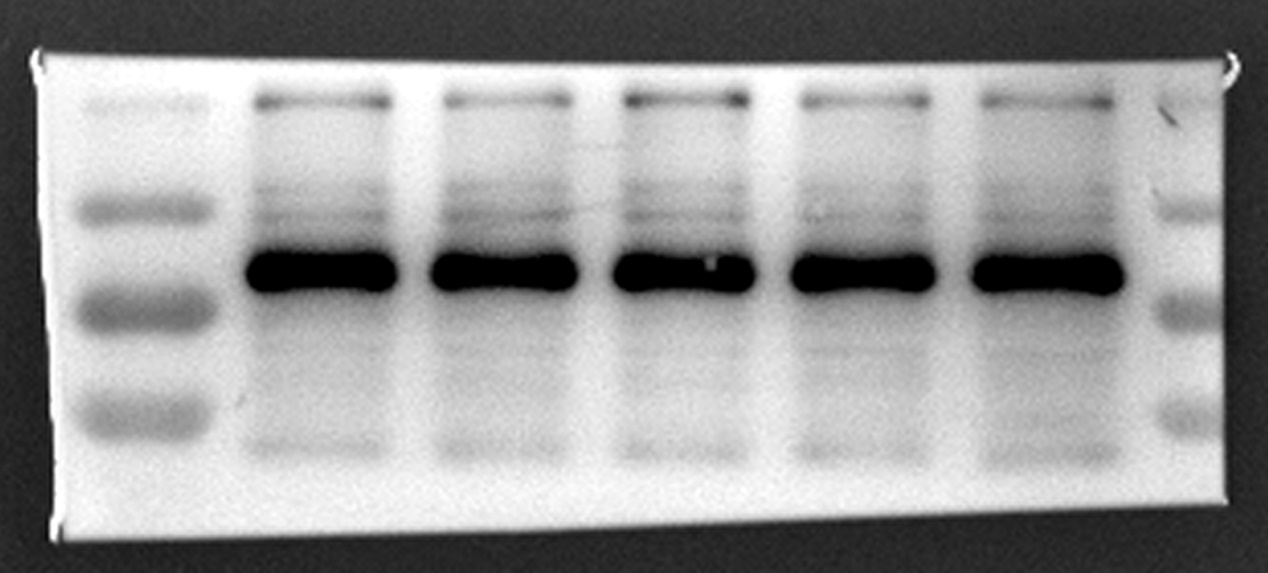


Smad3


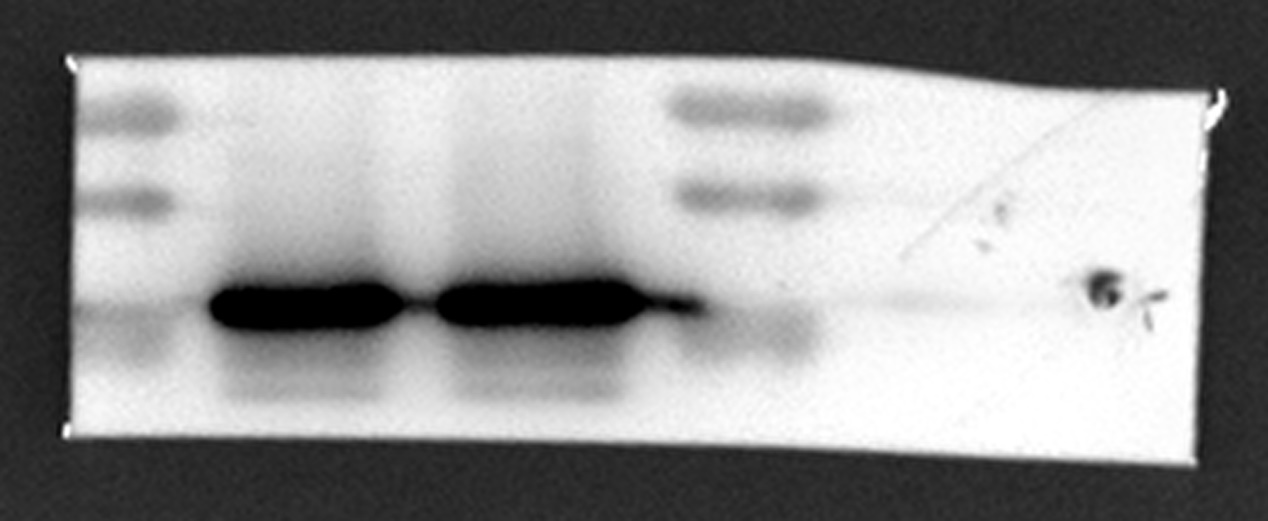


GAPDH


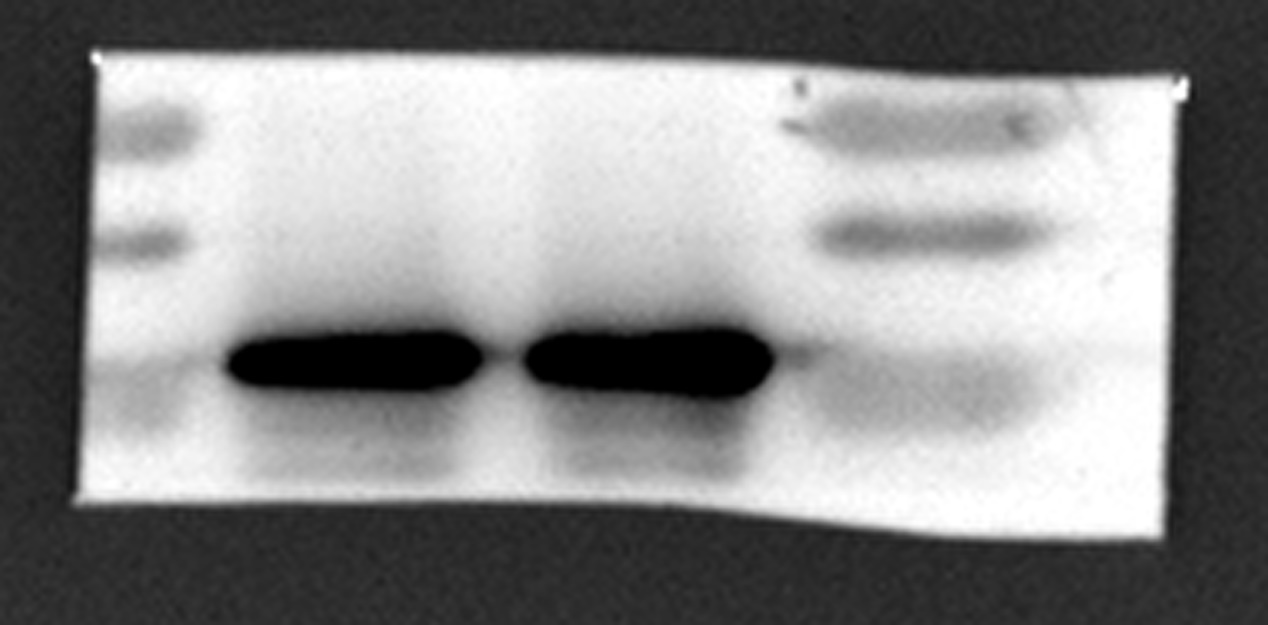


GAPDH


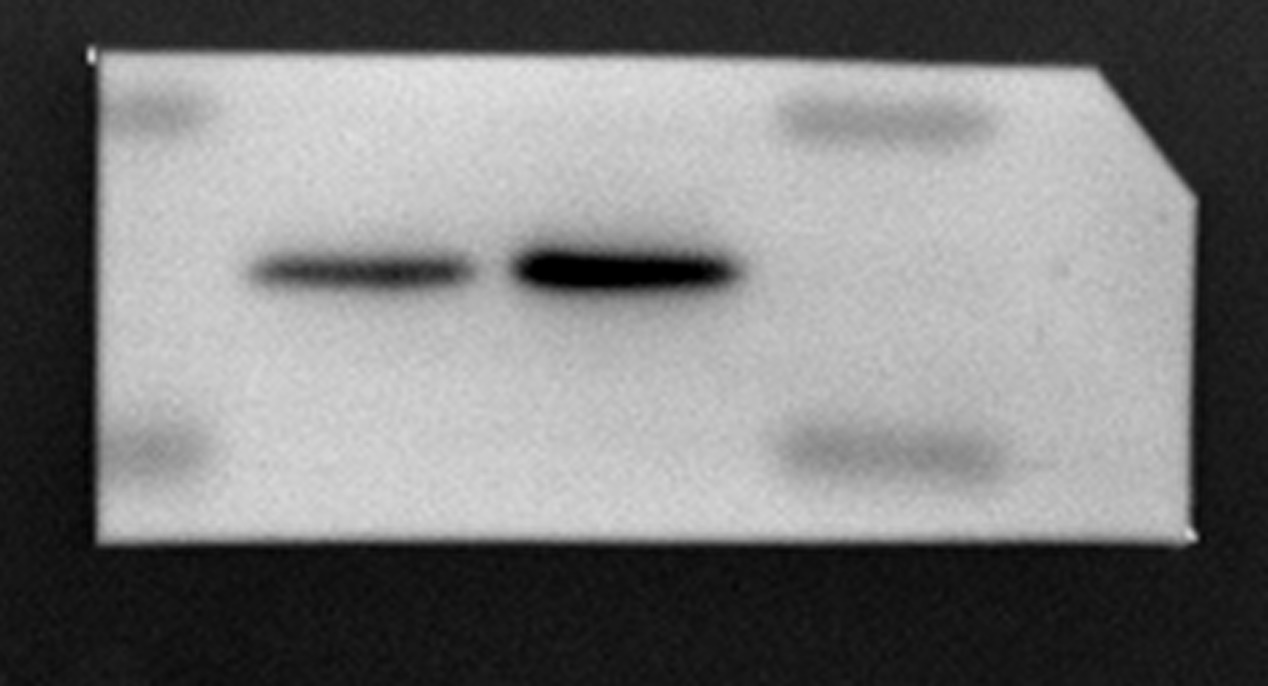


M1 receptor


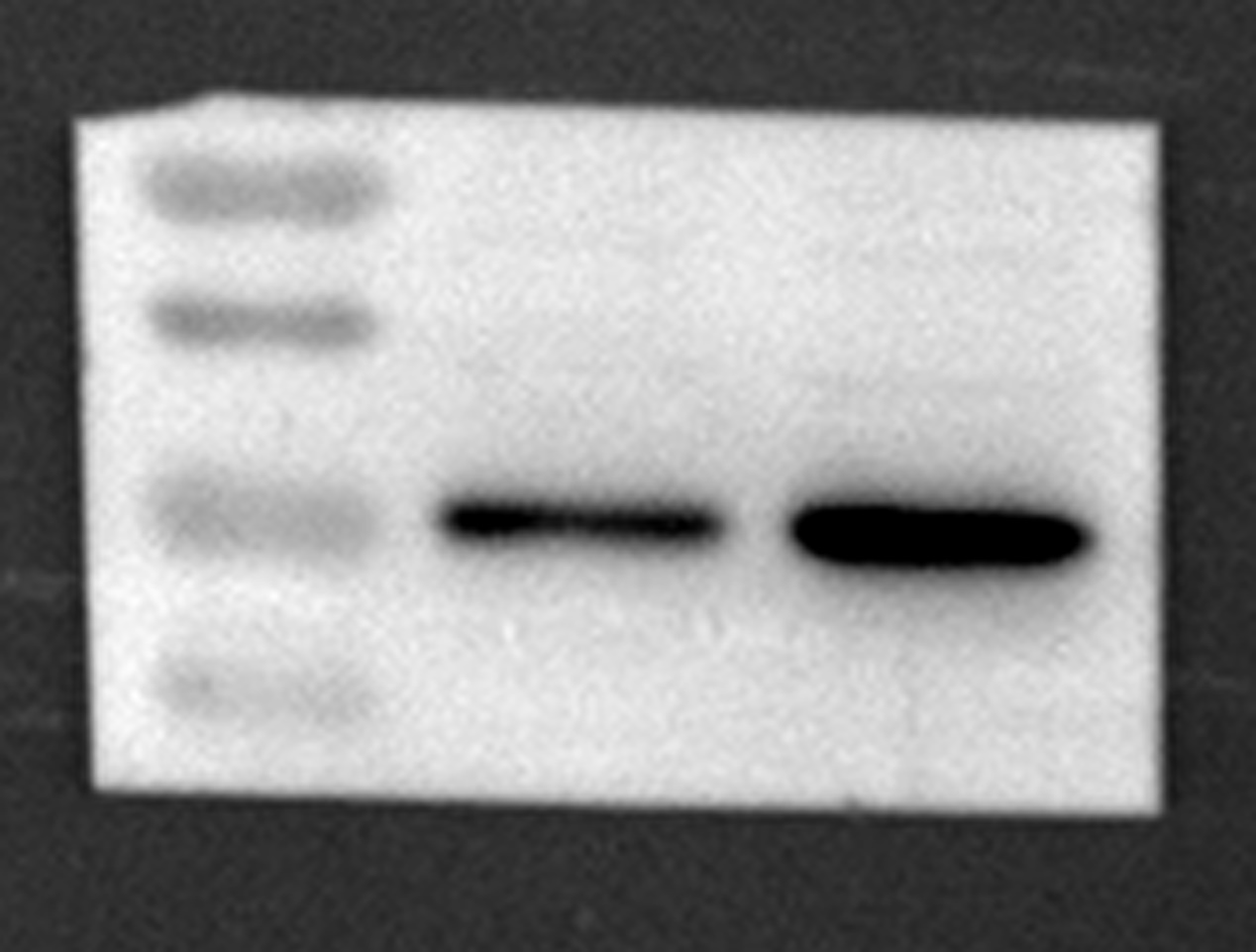


M2 receptor


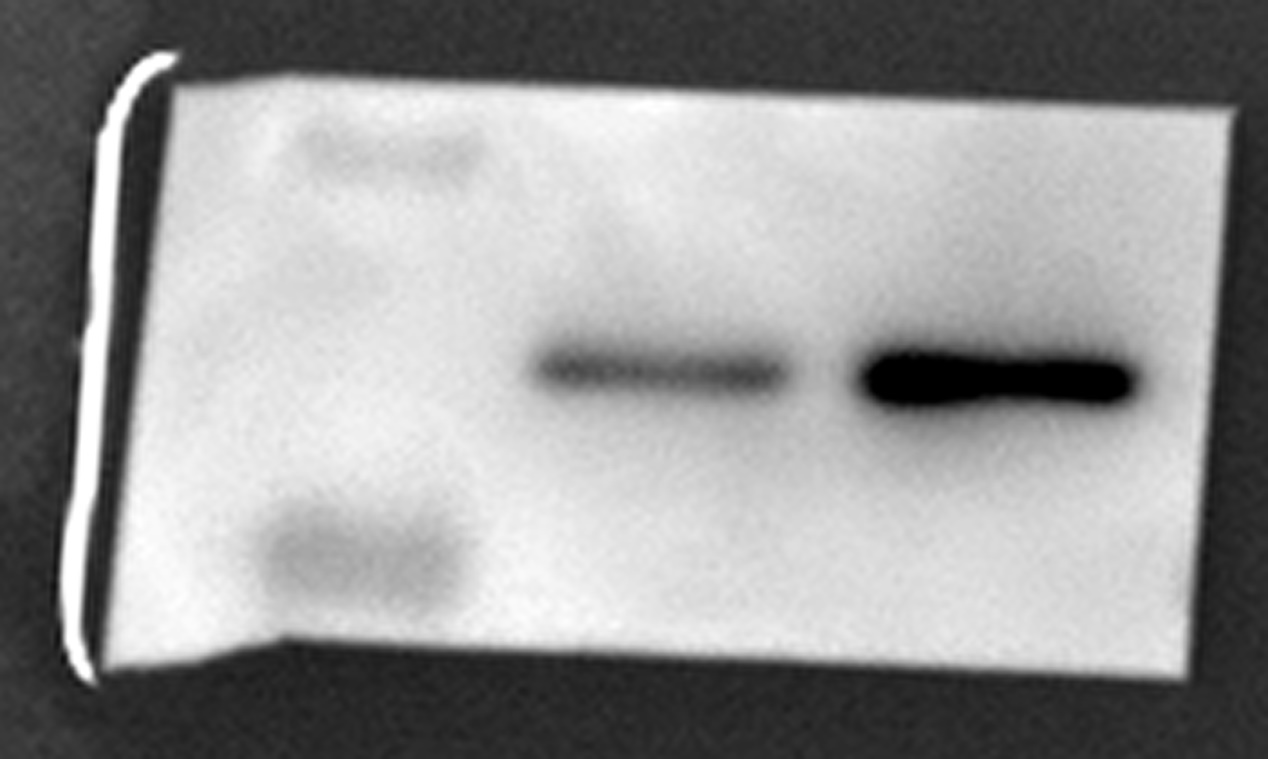


M3 receptor


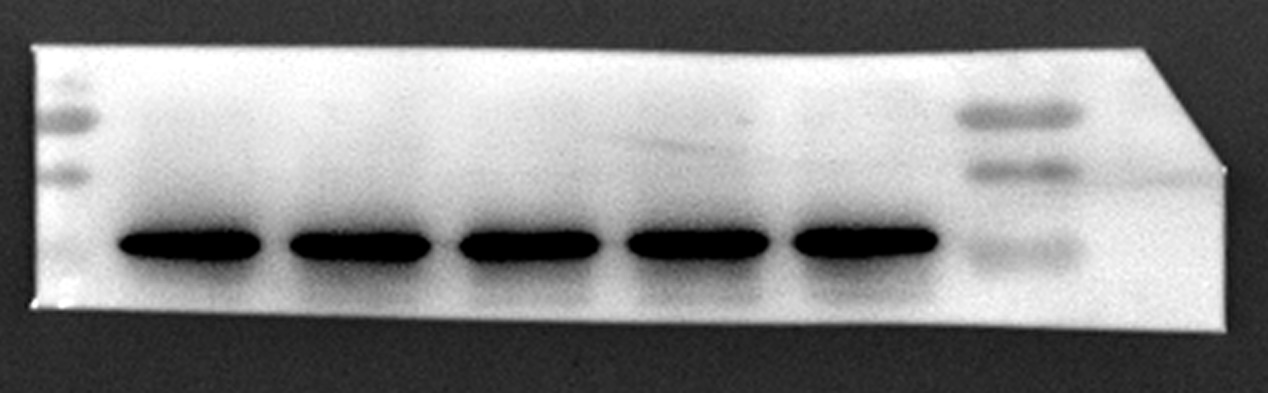


GAPDH


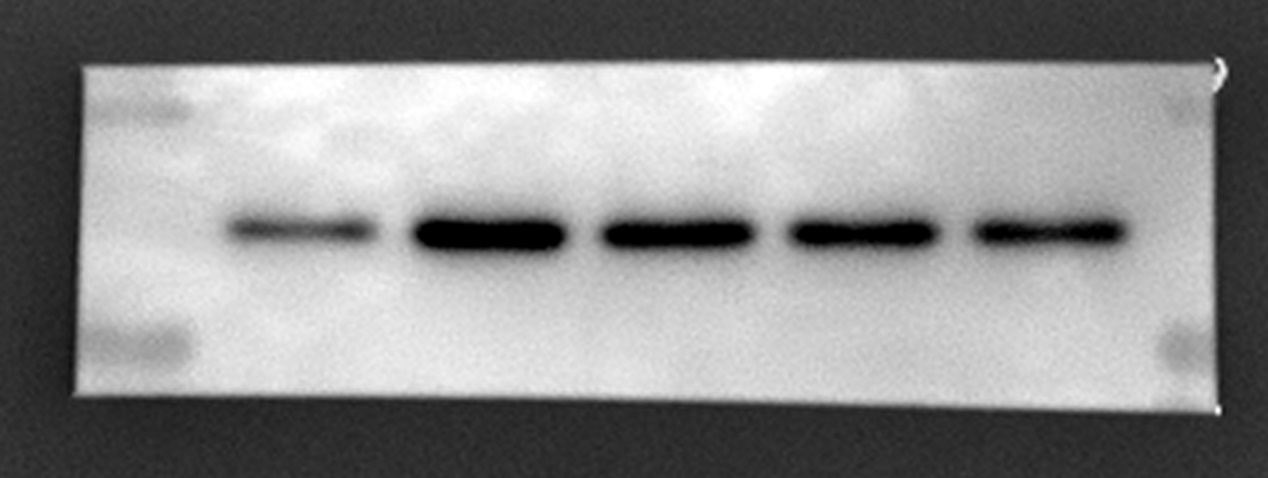


M3 receptor


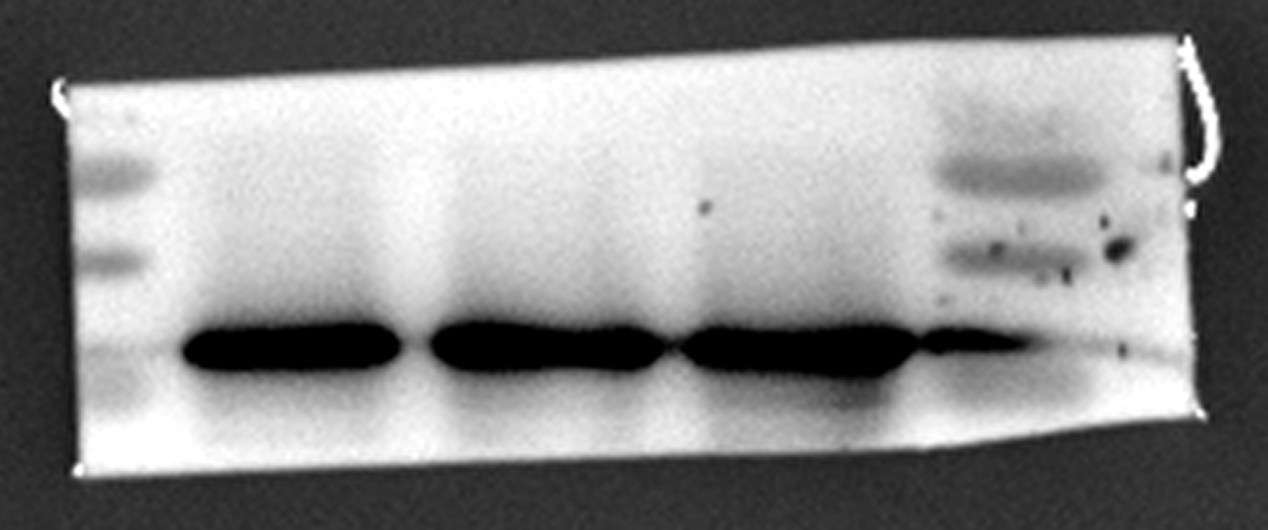


GAPDH


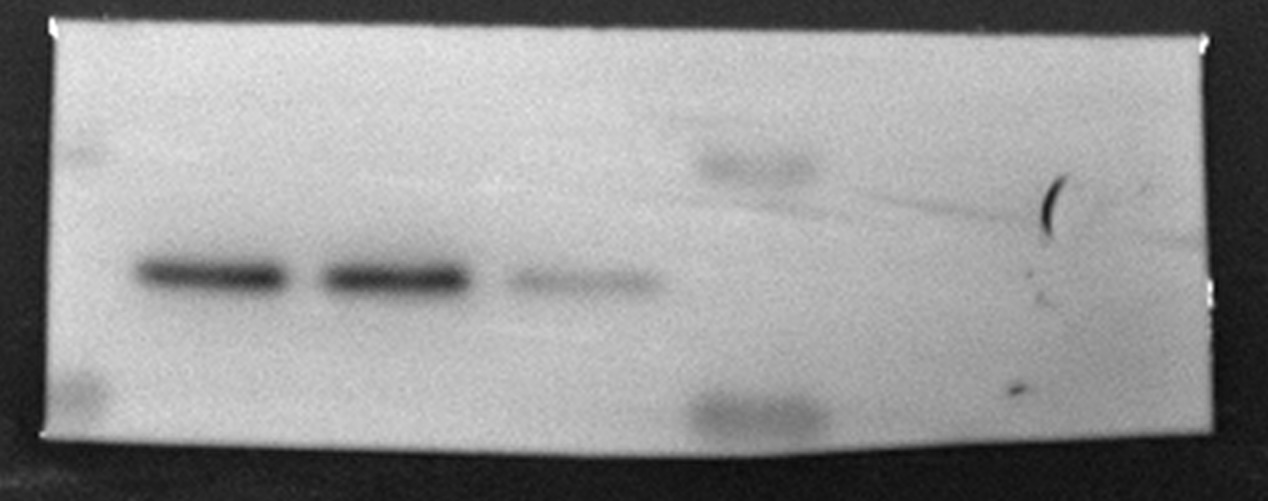


M3 receptor


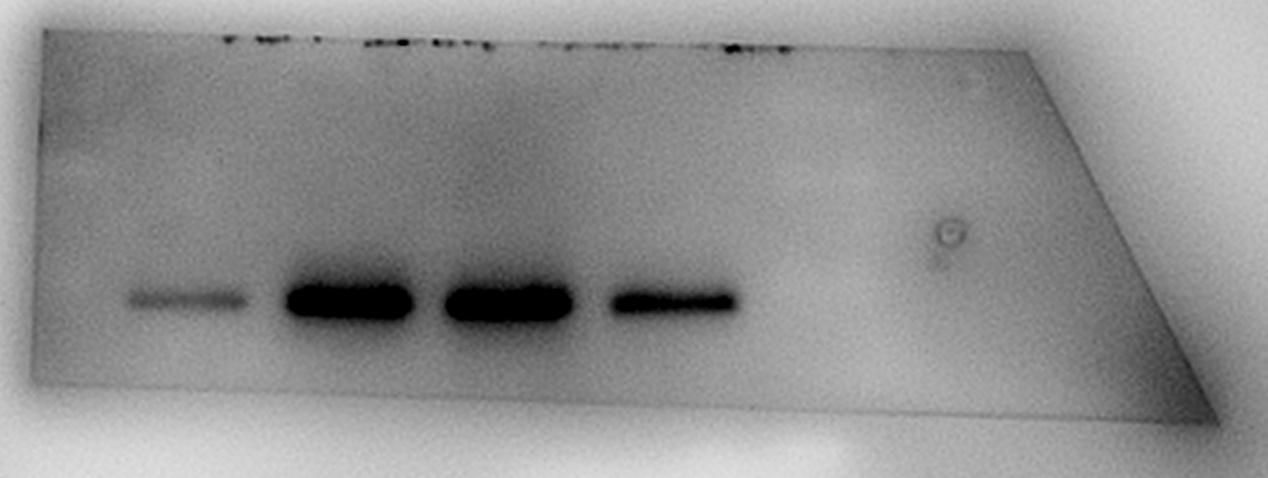


Col-Ⅰ


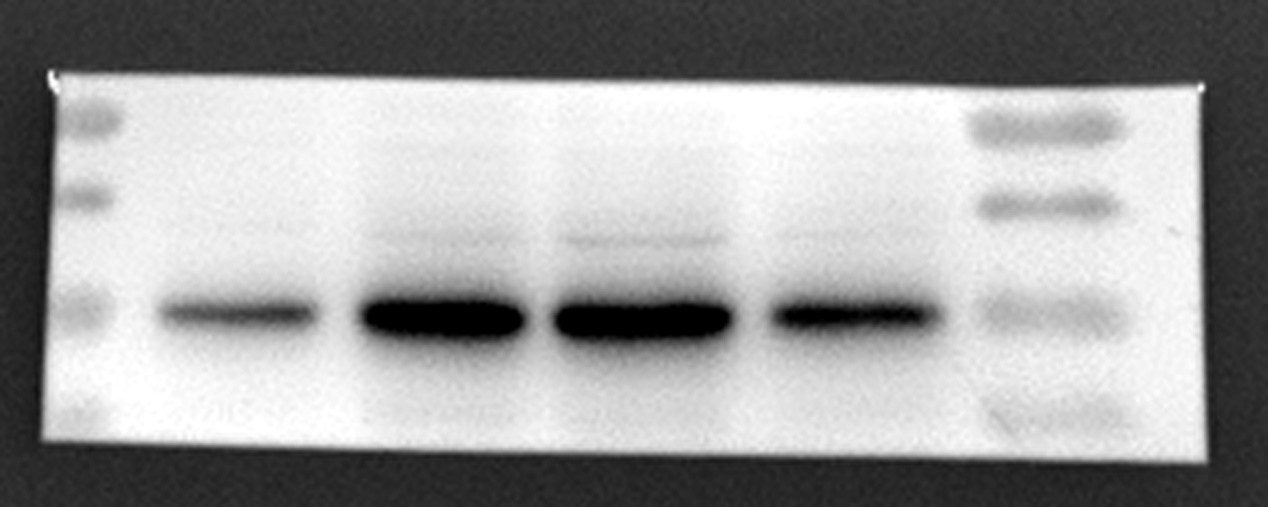


Col-Ⅲ


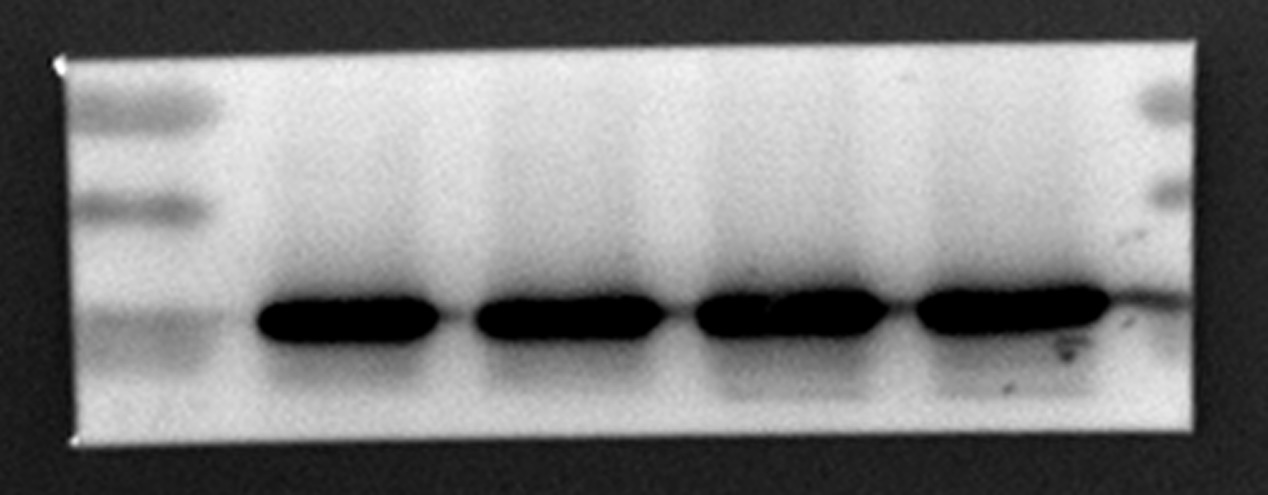


Gapdh


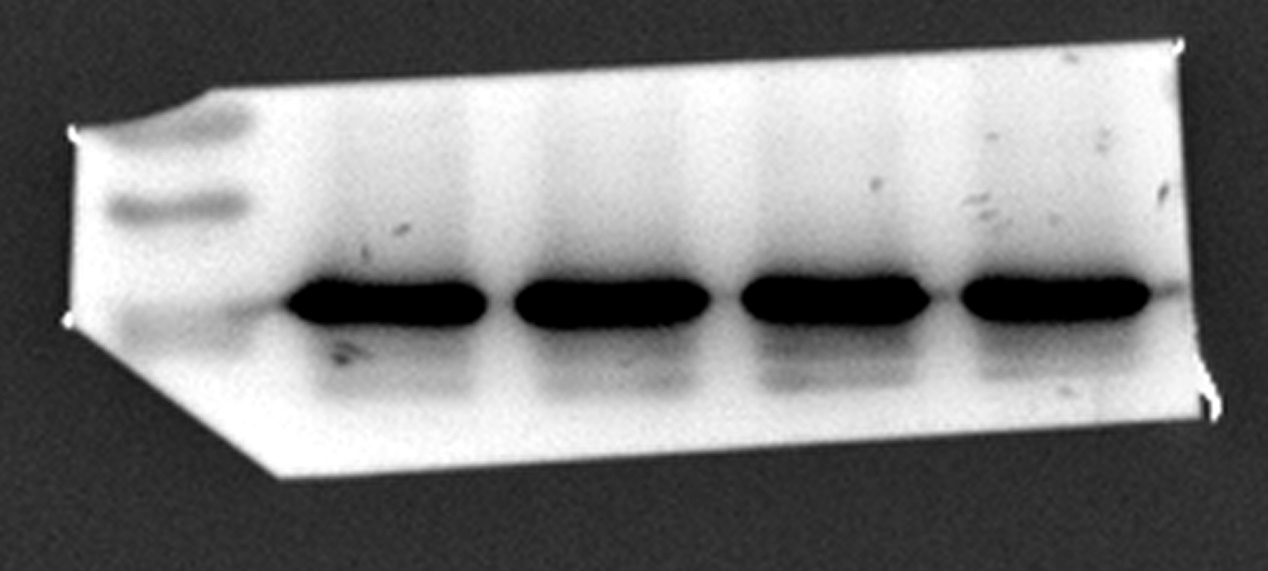


GAPDH


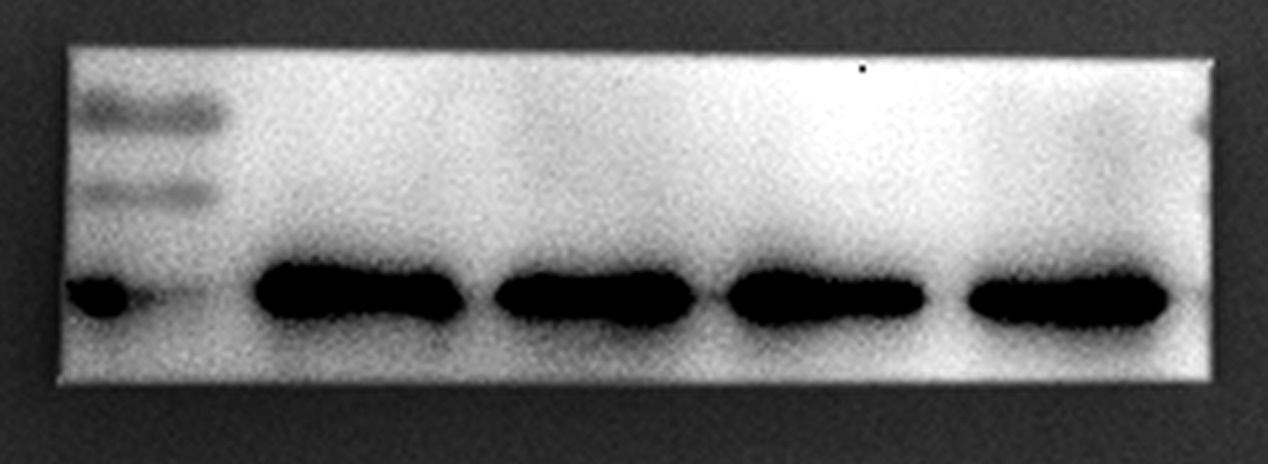


GAPDH


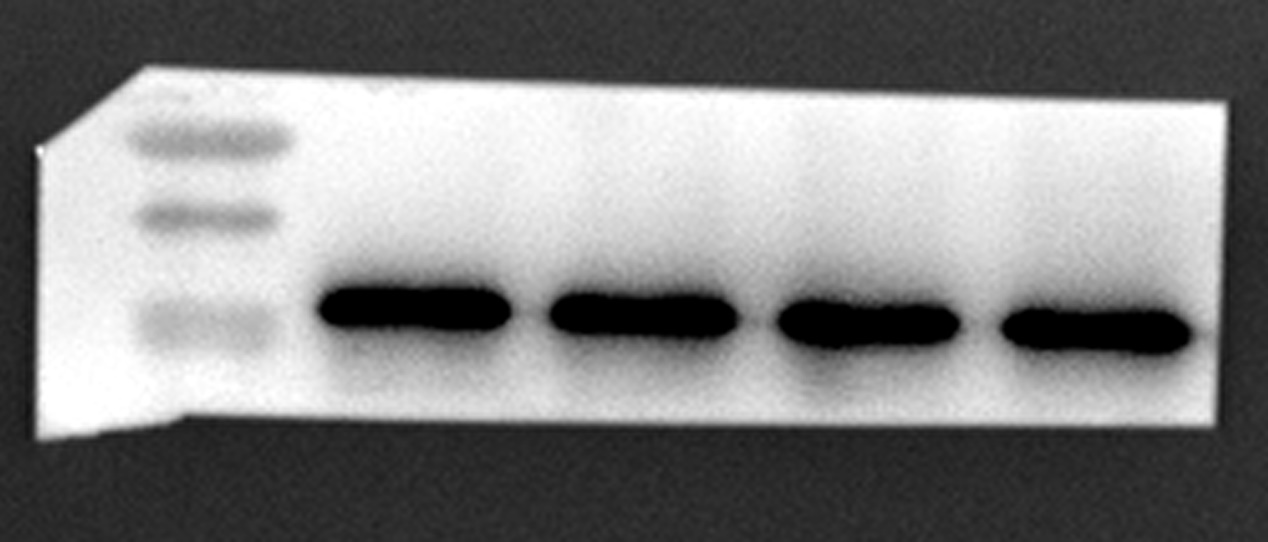


GAPDH


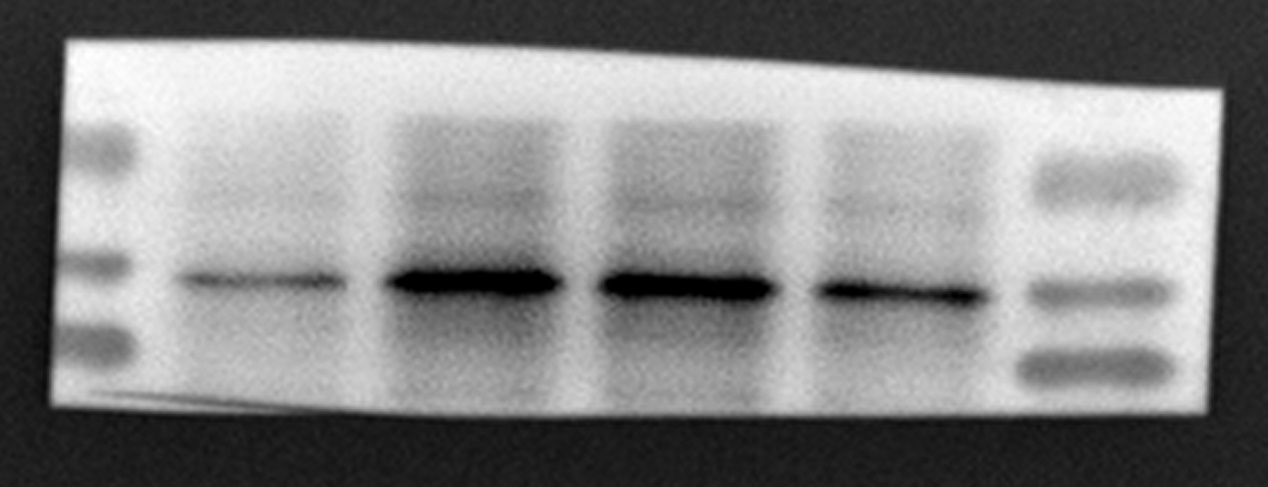


P-Smad2


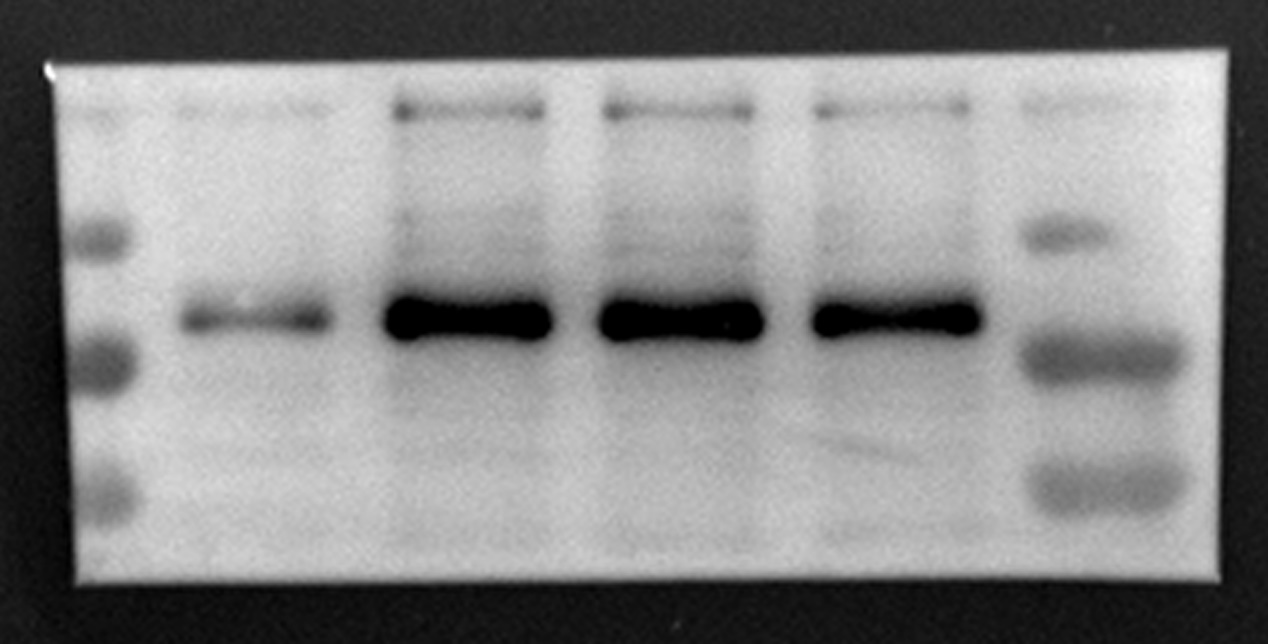


P-Smad3


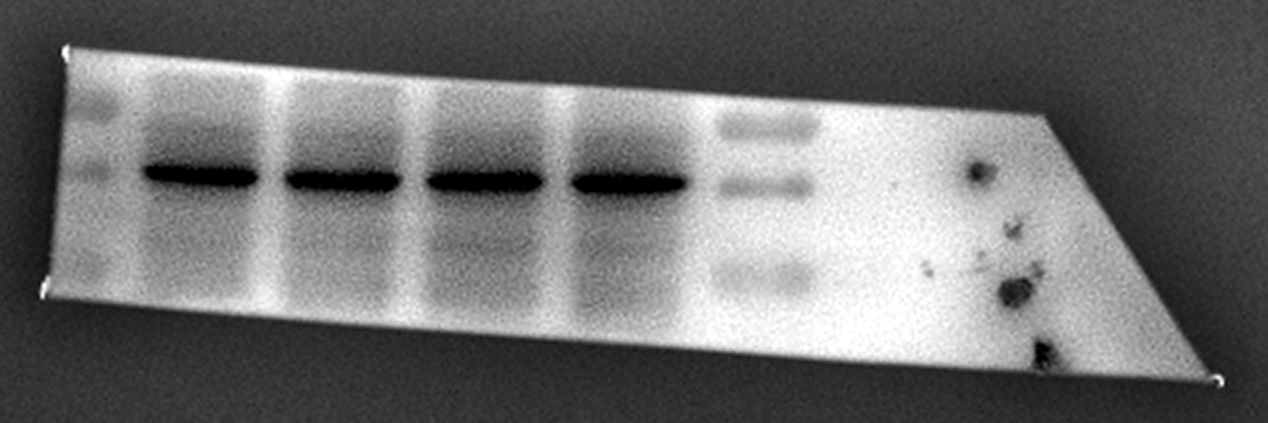


Smad2


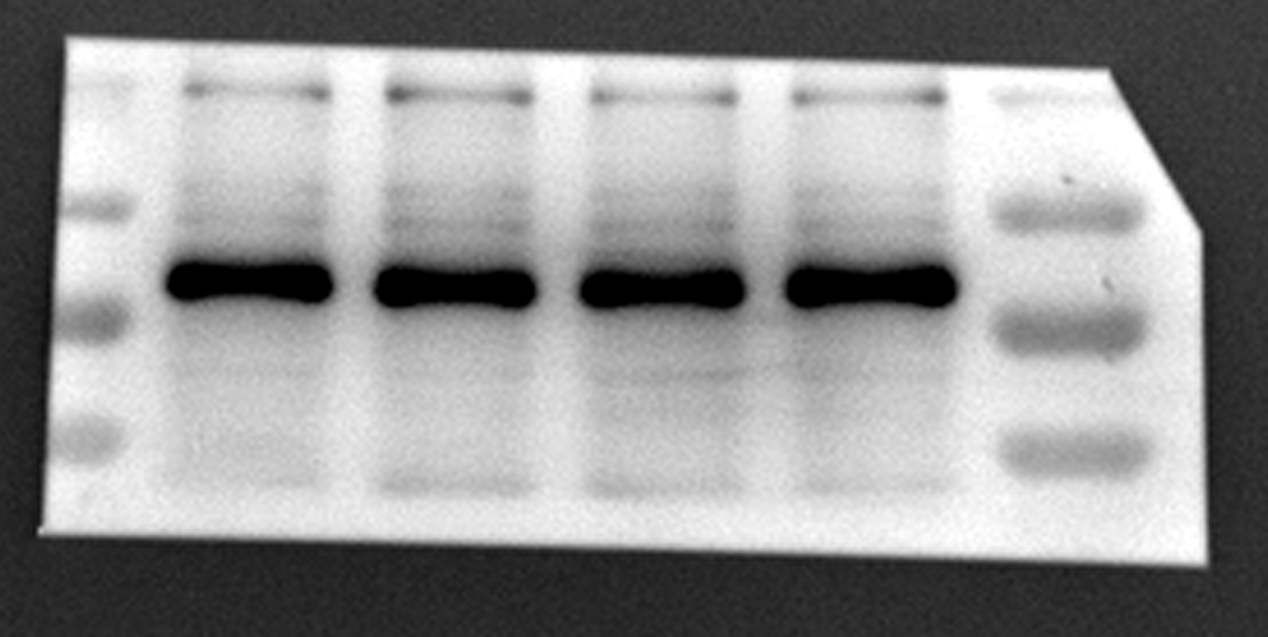


Smad3


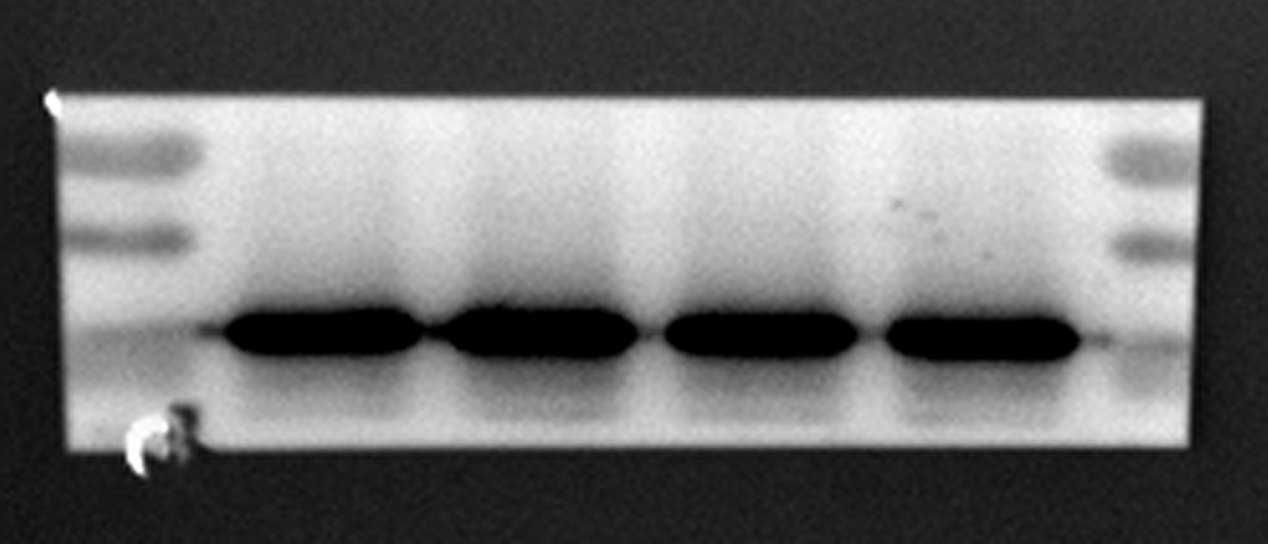


GAPDH


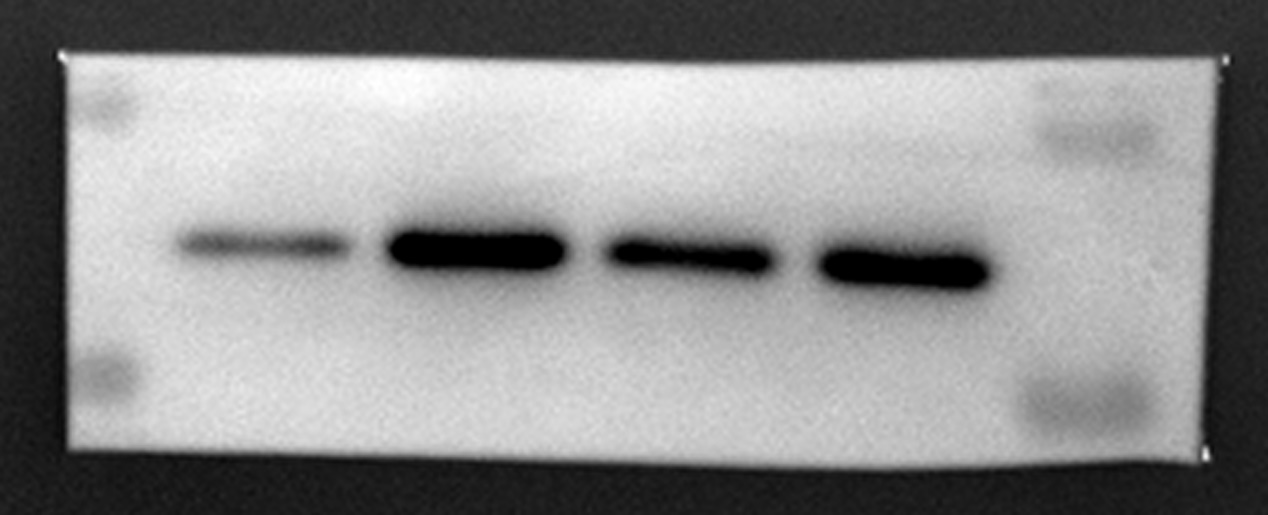


M3receptor


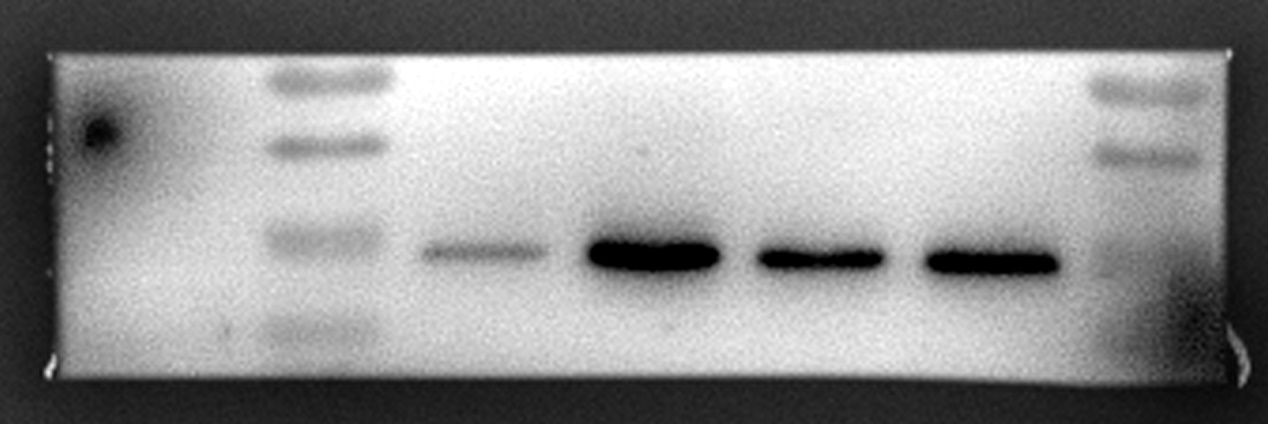


Col-Ⅰ


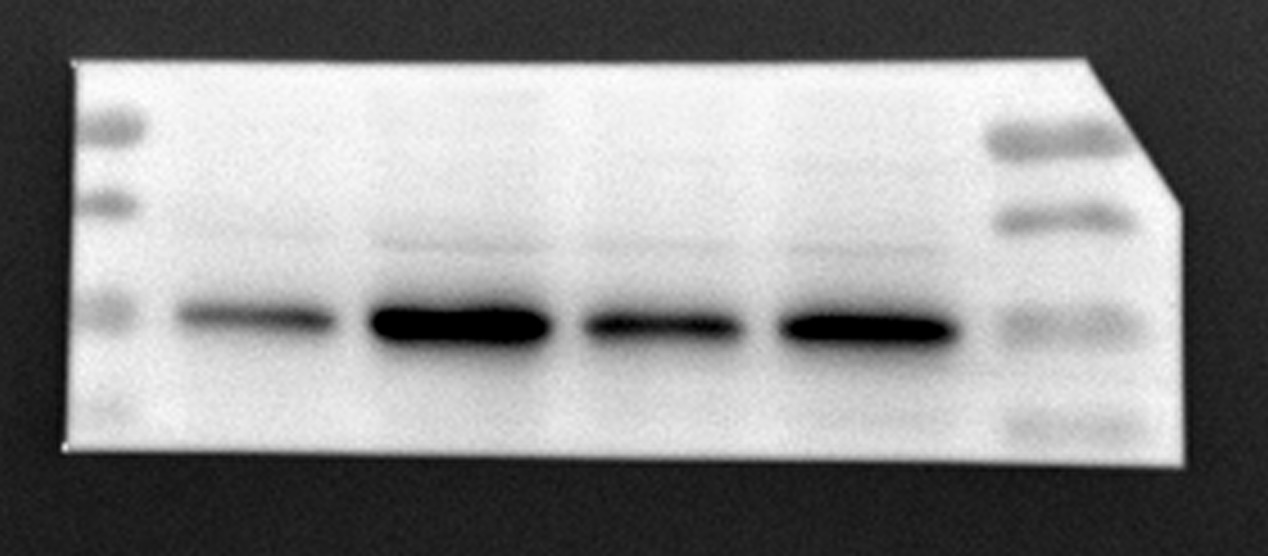


Col-Ⅲ


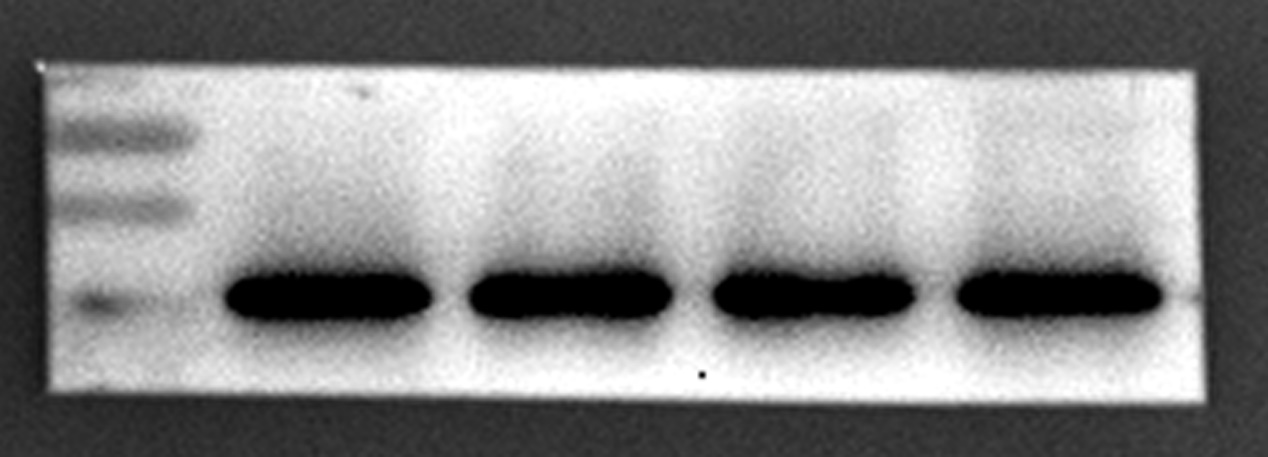


GAPDH


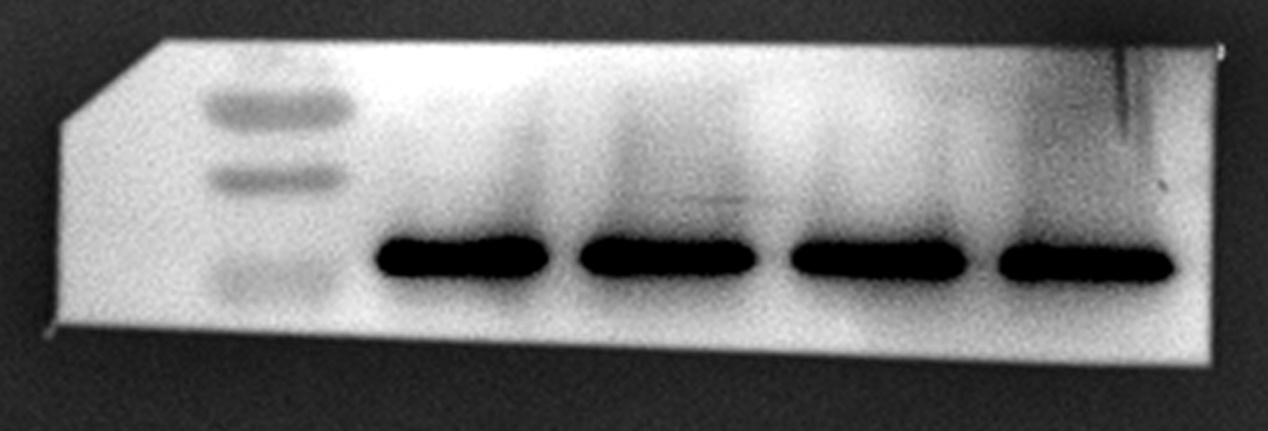


GAPDH


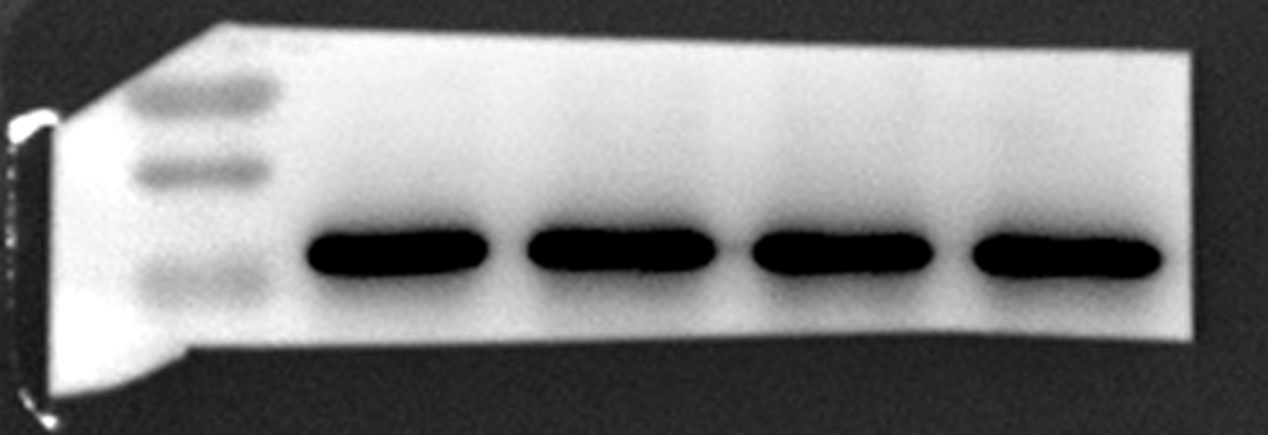


Gapdh


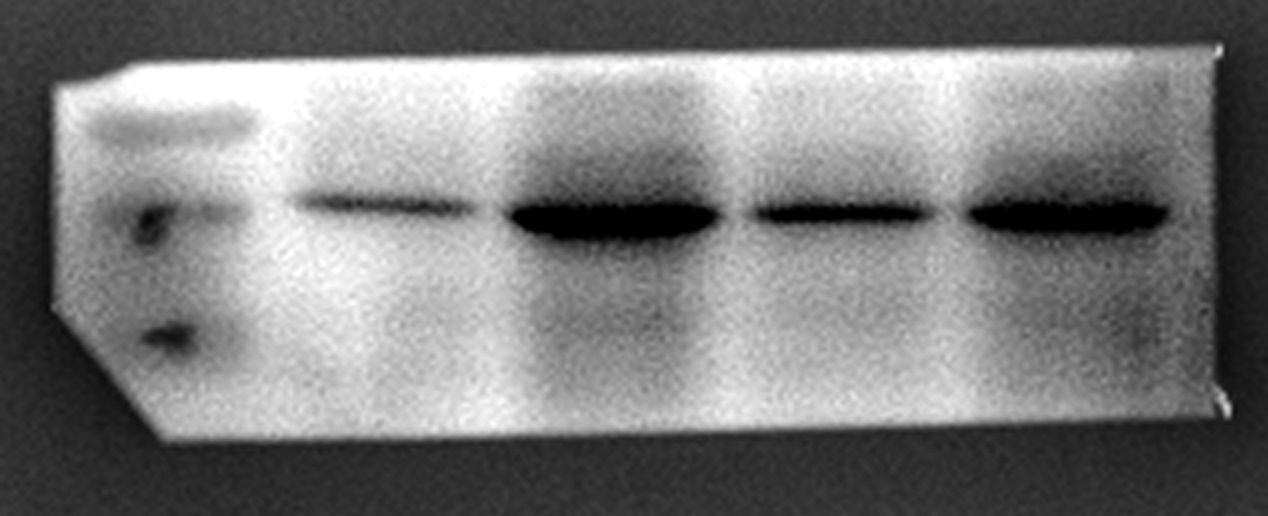


p-Smad2


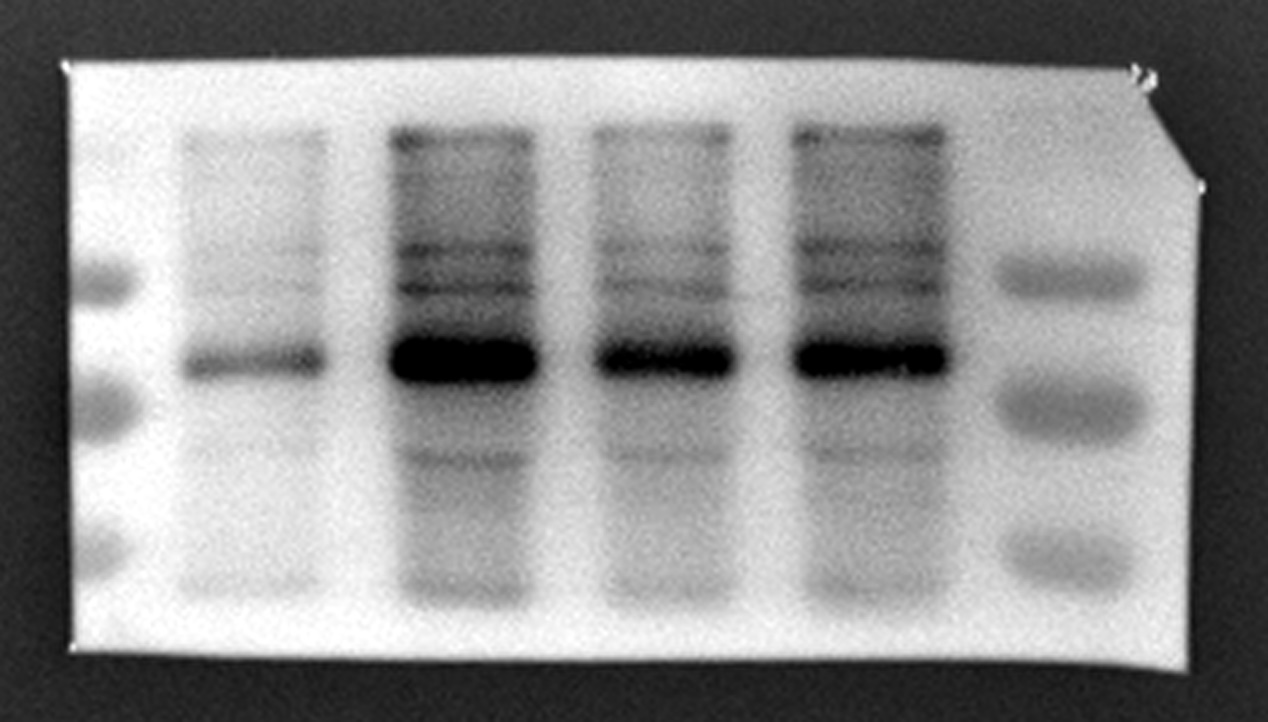


p-Smad3.


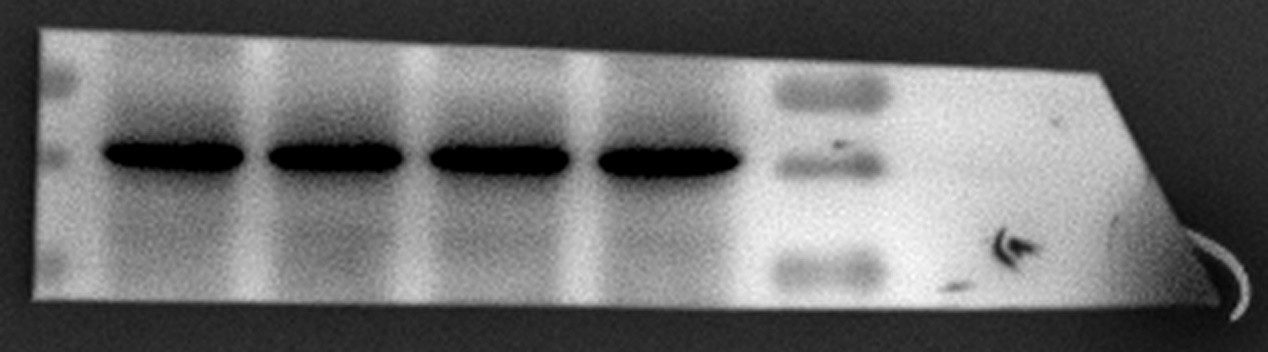


Smad2


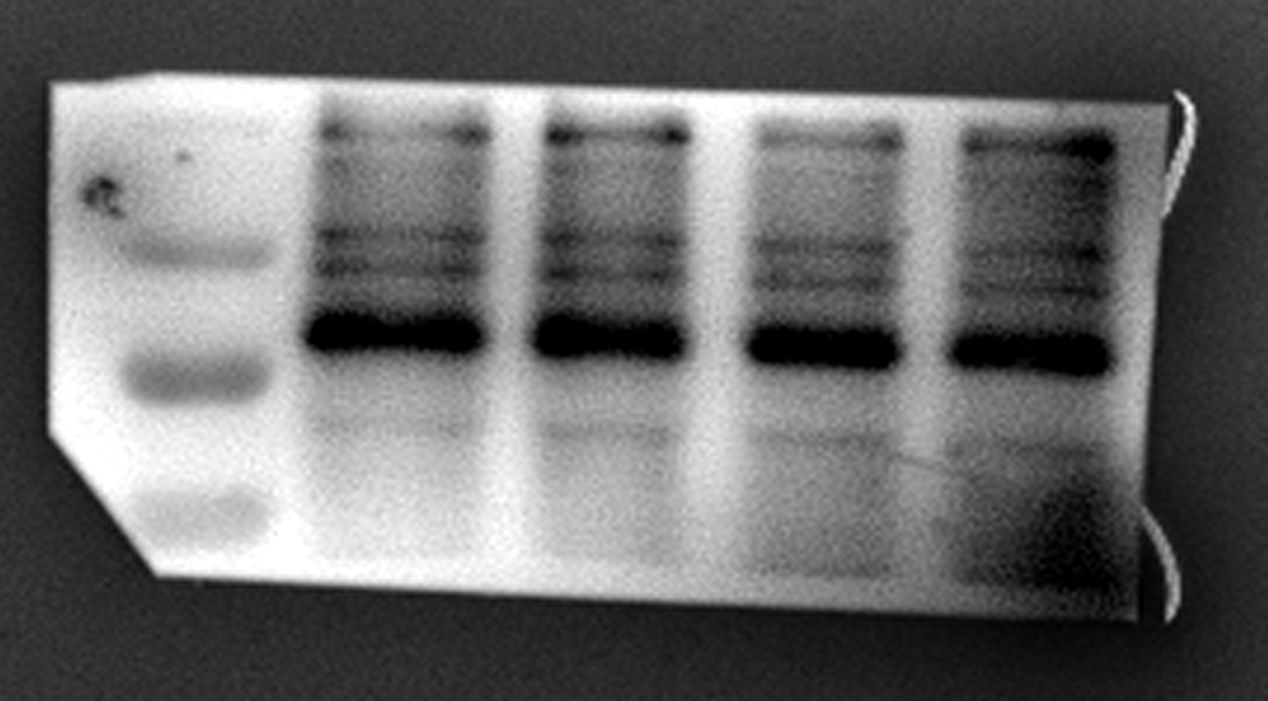


Smad3
